# The role of microbial ecology in improving the performance of anaerobic digestion of sewage sludge

**DOI:** 10.3389/fmicb.2022.1079136

**Published:** 2022-12-14

**Authors:** Christian Krohn, Leadin Khudur, Daniel Anthony Dias, Ben van den Akker, Catherine A. Rees, Nicholas D. Crosbie, Aravind Surapaneni, Denis M. O'Carroll, Richard M. Stuetz, Damien J. Batstone, Andrew S. Ball

**Affiliations:** ^1^ARC Training Centre for the Transformation of Australia's Biosolids Resource, RMIT University, Bundoora, VIC, Australia; ^2^School of Health and Biomedical Sciences, Discipline of Laboratory Medicine, STEM College, RMIT University, Bundoora, VIC, Australia; ^3^South Australian Water Corporation, Adelaide, SA, Australia; ^4^Melbourne Water Corporation, Docklands, VIC, Australia; ^5^Water Research Centre, School of Civil and Environmental Engineering, University of New South Wales, Sydney, NSW, Australia; ^6^Australian Centre for Water and Environmental Biotechnology, Gehrmann Building, The University of Queensland, Brisbane, QLD, Australia

**Keywords:** renewable natural gas, foaming, pollutant monitoring, microbial ecology, circular economy, biomarker, meta-omics, biosolids

## Abstract

The use of next-generation diagnostic tools to optimise the anaerobic digestion of municipal sewage sludge has the potential to increase renewable natural gas recovery, improve the reuse of biosolid fertilisers and help operators expand circular economies globally. This review aims to provide perspectives on the role of microbial ecology in improving digester performance in wastewater treatment plants, highlighting that a systems biology approach is fundamental for monitoring mesophilic anaerobic sewage sludge in continuously stirred reactor tanks. We further highlight the potential applications arising from investigations into sludge ecology. The principal limitation for improvements in methane recoveries or in process stability of anaerobic digestion, especially after pre-treatment or during co-digestion, are ecological knowledge gaps related to the front-end metabolism (hydrolysis and fermentation). Operational problems such as stable biological foaming are a key problem, for which ecological markers are a suitable approach. However, no biomarkers exist yet to assist in monitoring and management of clade-specific foaming potentials along with other risks, such as pollutants and pathogens. Fundamental ecological principles apply to anaerobic digestion, which presents opportunities to predict and manipulate reactor functions. The path ahead for mapping ecological markers on process endpoints and risk factors of anaerobic digestion will involve numerical ecology, an expanding field that employs metrics derived from alpha, beta, phylogenetic, taxonomic, and functional diversity, as well as from phenotypes or life strategies derived from genetic potentials. In contrast to addressing operational issues (as noted above), which are effectively addressed by whole population or individual biomarkers, broad improvement and optimisation of function will require enhancement of hydrolysis and acidogenic processes. This will require a discovery-based approach, which will involve integrative research involving the proteome and metabolome. This will utilise, but overcome current limitations of DNA-centric approaches, and likely have broad application outside the specific field of anaerobic digestion.

## Introduction

Anaerobic digestion (AD) of primary and/or secondary sewage sludge often occurs in stirred, anaerobic reactors. In the absence of oxygen, mixed microbial communities reduce sludge volume, mass, the biodegradable organic fraction, and pathogen load, with renewable natural gas a desired by-product of this process. There are strong economic and operational drivers to improve digestion performance and reliability. Fifty percent of the long-term operating costs of wastewater treatment plants (WWTPs) can be attributed to the management of sludge solids (Appels et al., 2008), with increasing demands for stringent discharge and emission requirements for operators. Hence, improving stability, energy and material recoveries, as well as enhancing monitoring capabilities of pathogens, antibiotic resistance genes (ARGs) or pollutants is important for sustainable long-term management of sewage wastes globally (Batstone and Virdis, 2014; Edwards et al., 2015; Ngo et al., 2021). The key to unlocking such improvements is a better understanding of the microbial capability, linked to population and functional diversity involved in AD.

**Figure 1:**
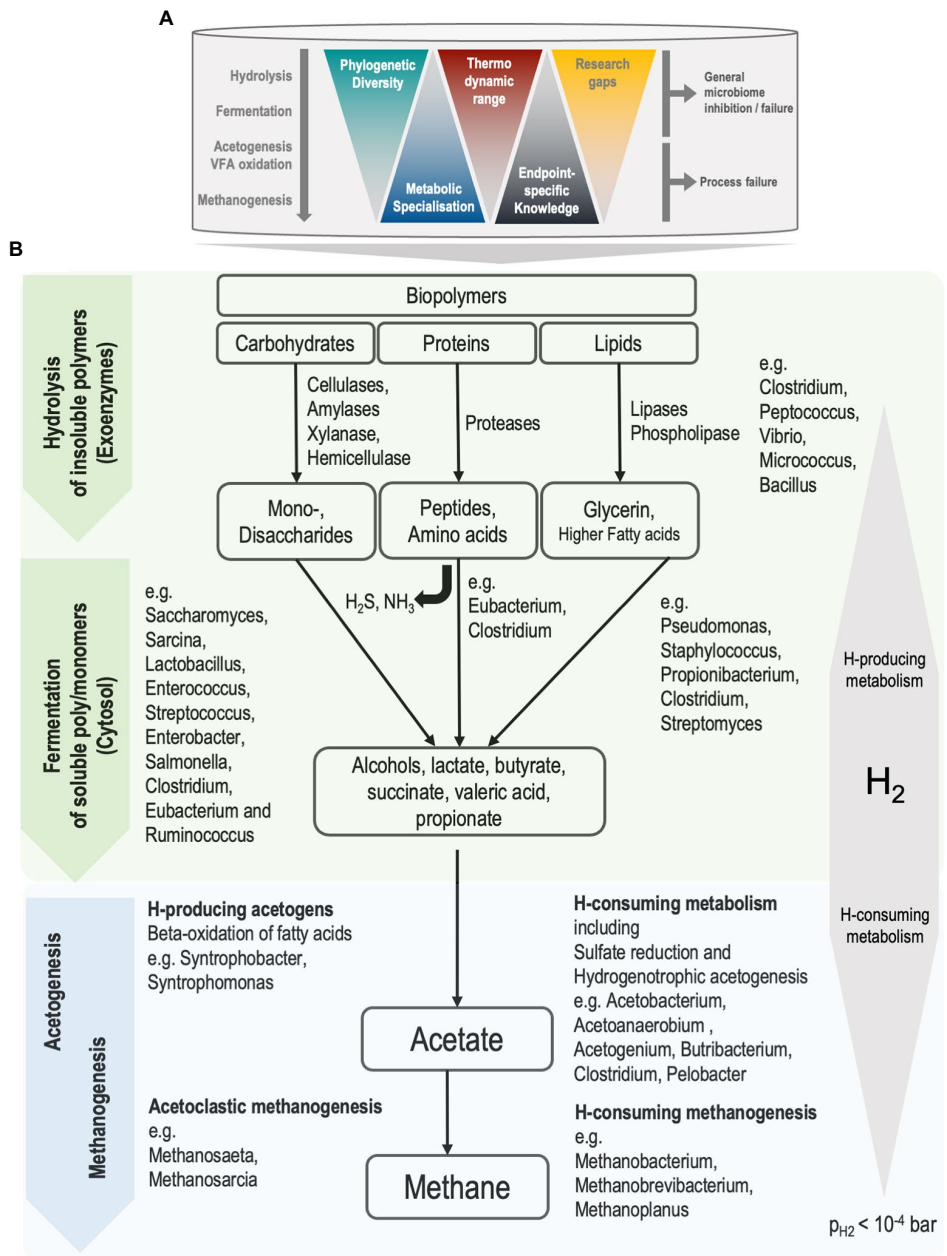
**(A)** The process of anaerobic digestion in mesophilic continuous-stirred tank reactors. Knowledge gaps are larger for biochemical processes that are facilitated by front-end organisms, the generalists. **(B)** Simplified representation of biochemical pathways during anaerobic sludge digestion and associated organisms. Front-end and back-end processes are represented as green and blue, respectively. Partial pressure of hydrogen (pH_2_) below 10^−4^  bar is required for favourable thermodynamics of syntrophic metabolism to drive reactions towards methane production. Ammonia (NH_3_) and hydrogen sulfide (H_2_S) are produced during the fermentation of amino acids.

The major anaerobic metabolic pathways leading to the conversion of organic materials to biogas are well established (Batstone et al., 2002a; Anderson et al., 2003; Gerardi, 2003; Batstone, 2006; Rosenwinkel et al., 2015; Meegoda et al., 2018). Anaerobic digestion is considered a structured metabolic process, with a frontend and backend, where the frontend refers to hydrolysis and fermentation, and the backend refers to volatile fatty acid (VFA) oxidation to methanogenesis. The backend is dominated by obligate specialists, has limited diversity, and is relatively well understood in the context of AD performance, physiochemical processes, rate-limitations, inhibitions or toxicities (Batstone and Jensen, 2011). In contrast, the frontend has a wide range of ecological diversity, with a high degree of redundancy in candidate organisms. Indeed, the diversity involved in AD can be seen as an inverted triangle. Most phylogenetic diversity is at the top, while our operationally relevant metabolic and community understanding lies mainly at the bottom of the triangle, with more knowledge available of the thermodynamically constrained metabolism of methanogens (Figure 1). At the top, comparatively little knowledge is available of fermenters and hydrolysers that metabolise a wider range of carbon and energy sources. This is particularly important in processes where hydrolysis is rate limited, such as sludge digestion (Batstone and Jensen, 2011). Hence, the potential for improving reactor performance lies in uncovering causes of poor energy conversion or process failures that involve interactions within the front-end metabolism.

The latest sequencing technologies have advanced to a point that make it possible to monitor complex microbial populations in near real time. The limitation to apply these technologies in the water sector lie only in the ability to analyse the vast amount of data generated from such technology in a meaningful way. Extracting biological patterns and associations of community interactions out of sludge systems will take time but will ultimately lead to next generation biomarkers of AD (De Vrieze, 2020). In biomedical research, the use of biomarkers to infer endpoints is common (Strimbu and Tavel, 2010). From research on the human microbiome it is evident that it can provide disease markers with greater confidence than even the human genome itself (Gilbert et al., 2018).

The application of ecological markers in AD is presently hampered by a lack of models that link relevant metabolic processes to the sludge biome. This is partly due to a high degree of numerical uncertainty originating from incomplete reference databases, multiple sources of methodological bias and ongoing challenges for normalisation of sparse, compositional sequencing data (McMurdie and Holmes, 2014; Gloor et al., 2017; Soppa, 2017; Louca et al., 2018; McKnight et al., 2019; Pérez-Cobas et al., 2020). As a result, some authors recommend approaches that seek numerical agreement from multiple methods in microbiome studies (Nearing et al., 2022). Furthermore, microbial ecologists have started exploring community-level functional traits, as opposed to simply comparing individual species or taxonomic compositions, which we will explore further in this review (Wood and Franks, 2018; Bittleston et al., 2021).

Developing biomarkers, inevitably involves ecology, the study of interactions between organisms and their environment (Ganong, 1904), which provides the principles and frameworks for investigations and marker discovery. As McMahon et al. (2007) argued, the development and integration of ecological principals into a predictive framework will enable the exploitation of engineered systems. One prominent example is the concept of the coupling of biodiversity with process stability, whereby increased biodiversity has been shown to improve digester performance and methanogenic recovery following disturbance (Briones and Raskin, 2003; McMahon et al., 2004; Cook et al., 2006).

With a focus on mesophilic anaerobic sewage sludge in continuously stirred reactor tanks, this review provides perspectives on the role of ecology in improving digester performance in WWTPs, as well as on the integration of next-generation tools available for a ‘systems biology’ approach. As such, it introduces trait-bases approaches and discusses the potential development and operational applications of ecological AD markers, derived from methods, such as targeted or untargeted sequencing or metabolomics. By describing the role of diversity and quantitative ecology, the review explores how to leverage existing knowledge to improve the process-performance of AD, particularly in preventing foaming or in improving biogas yields, pathogen reduction and pollutant monitoring.

## Anaerobic digestion of sewage sludge - *status quo* and research gaps

The principal biochemical pathways from hydrolysis to methane production are well described, and for simplicity, grouped into four to six different stages, each mediated by a distinct microbial clade. These stages, reviewed by Anderson et al. (2003) and Rosenwinkel et al. (2015), are represented in the Anaerobic Digestion Model No 1 (ADM1; Batstone et al., 2002b) and further illustrated in Figure 1. Generally, once the sludge mix (primary and waste-activated) enters the anaerobic and continuously stirred-tank reactor (CSTR), a complex ecosystem of microbial groups utilise any biodegradable carbon and energy sources, resulting in the decomposition of carbon mass to simpler compounds (ultimately C1 methane and carbon-dioxide). Importantly, in this closed system, many anaerobic processes (particularly energetically constrained oxidation/reduction reactions) are closely coupled and interdependent (Morris et al., 2013). Hence, disturbances to the sludge habitat are susceptible to process inhibition and failures, including potentially irreversible digester ‘souring’, whereby methanogenic activity stops due to the accumulation of organic acids. However, while the back-end organisms involved in VFA oxidation to methanogenesis are principal determinants of process stability, the frontend (particularly hydrolysis) is a key determinator of process performance (Batstone and Jensen, 2011). The degree to which the frontend is impacted by process conditions is largely unknown.

Acetogenesis and methanogenesis processes during AD were comprehensively documented (Batstone and Jensen, 2011). The wastewater sector has greatly advanced the process-relevant understanding of microbial and chemical processes for operators, enabling more effective mass balancing, and the expansion of anaerobic technologies. Detailed work from the 1970s, including on pure culture work has allowed strong understanding of niche-specific acetogens and methanogenic archaea in their core functional modes, leading to reference texts for Methanogens (Ferry, 1993) and Acetogens (Drake, 1994) which cover metabolic function, including limitations in detail. Poor process stability (and performance indicated by high organic acids) is commonly assessed as a quantity of endpoint activities, such as low specific acetoclastic methanogenic activity as proposed by Astals et al. (2015). These measurements reflect biostatic (reversible) and biocidal (irreversible) inhibitions, including those induced by acid overloading, hence are reliable indicators for process performance. That is because specialised organisms, such as acetogens and methanogens have narrow environmental niches, and are therefore sensitive to changes in sludge habitat (pH, temperatures, feedstock, toxins), leading to decreases in biogas production (Theuerl et al., 2019). Common inhibitors and processes leading to such failures and decreased biogas production have been reviewed (Chen et al., 2008; Theuerl et al., 2019) and are monitored with available methods and corrected by changing the operating point.

In contrast, activities of anaerobic generalists, such as bacterial hydrolysers, acidogens and acetogens and their role in inhibiting this engineered process are more complex and less understood (Figure 1). Activities of hydrolysers and fermenters may lead to broader microbiome inhibitions and failures, which may be the source of overall poor hydrolysis and energy conversion or associated with foaming events. While the principle metabolism of protein and sugar digestion is well understood (Batstone and Jensen, 2011), the actual pathways, regulating mechanisms, metabolism, and principal actors can vary widely, even for model substrates such as glucose (Hoelzle et al., 2021). Hydrolytic enzyme diversity, activity, and controlling mechanisms are poorly understood, particularly on complex substrates such as wastewater sludges. Batstone et al. (2019) noted that our current knowledge of anaerobic sugar and amino acid metabolic pathways has little predictive value in metabolic modelling for complex systems.

Despite a strong understanding of core functional metabolism in methogens, there is still significant discovery involving archaea, including direct electron transfer with bacteria for example, which is further explored in the next section. Overall, the knowledge gaps that prevent the development of quantitative, microbe specific models for AD include the types of relationships (linear or non-linear) between different types of organisms and their environment over time (Perez-Garcia et al., 2016). These research gaps may relate to (1) the complexity of the anaerobic microbiome and the large number of unknown species, functions and interactions; (2) the high degree of thermodynamic constraints in a continuous flow system requiring both, a highly adapted and specialised metabolism, while also requiring a high diversity and therefore resilience for process stability and (3) the high degree of heterogeneity between digesters globally, due to variability in feed, reactor configuration and operational conditions, and in organism types that enter the anaerobic digester with sewage sludge.

## Baseline ecological observations

Microbial communities in anaerobic digesters are dynamic and respond quickly to environmental changes in the reactor (De Vrieze et al., 2013). There are likely to be four overlapping and interlinked ‘general drivers’ of microbial change in an AD reactor: [1] Seasonal forcing factors (e.g., temperature changes), [2] event-based forcing (e.g., large rainfall events; inflow of a toxicant), [3] operator forcing (changes imparted by decisions made by the plant operator) and [4] stochastic events. As a result, the individual reactor environment determines the overall microbial function and performance (Vanwonterghem et al., 2014a; Peces et al., 2018).

Predicting reactor performance quantitatively from the presence of identifiable microbial taxa has proven to be difficult, mainly due to two reasons. Firstly, microbial functional traits, which influence reactor performance, can be inherited by taxonomically unrelated microorganisms, especially if these are widely shared (e.g., glycolysis; Martiny et al., 2013). Secondly, it is still unknown what around ~50% of the most abundant genera are accomplishing (Nierychlo et al., 2020a), with a large proportion of these organisms apparently immigrating from the sludge feed and not growing or participating in anaerobic processes (Kirkegaard et al., 2017; Jiang et al., 2021b). The abundance of some of the most specialised genera, such as the acetoclastic *Methanothrix* (formerly *Methanosaeta*) or the acetoclastic/hydrogenothrophic *Methanosarcina* provide reliable process (i.e., methane production) and stability indicators (Ho et al., 2013; Carballa et al., 2015). Shifts in the methanogen population away from *Methanosaetaceae* have been observed during foaming (He et al., 2017), with possible drivers for this shift being either the conditions leading to foaming, or the mechanical selection process of foaming itself (i.e., removal of filamentous *Methanosaetaceae*). However, without knowing the impacts of a large pool of unknown hydrolytic and fermentative activities, it seems impossible to robustly predict downstream process performance.

Furthermore, it is apparent that we are just beginning to capture the full functional diversity of prokaryotes, as recent developments revealed an enormous diversity within the ancient methanogenic pathways, resulting in a range of discoveries of archaea and a complete re-classification of the phylum *Euryarchaeota* in the Genome Taxonomy Database (Adam et al., 2017; Lyu et al., 2018; Berghuis et al., 2019; Rinke et al., 2021). For example, a metatranscriptomics study from 2014 discovered a completely new methane-producing pathway of *Methanothrix* species. Instead of utilising acetate, the study found that it reduced carbon dioxide to methane *via* direct electron transfer from *Geobacter* during ethanol transfer (Rotaru et al., 2014). The discovery of this direct interspecies electron transfer (DIET) has broadened the ecological range for methanogens, particularly those previously regarded as obligate or specialist acetoclastic organisms such as *Methanothrix* and *Methanosarcina* (Lovley, 2017). Other such discoveries are expected, given the sizeable knowledge gaps in the upstream metabolism of anaerobic sludge. Currently, the fundamental advantage of DIET in enhancing methanogenesis over molecular interspecies electron transfer is a subject of debate (Desmond-Le Quéméner et al., 2021).

The global core microbiome, which defines the most abundant and shared species across different anaerobic reactors, is composed of relatively few species. Amplicon sequencing, which is used for most taxonomic assessments, is typically not sensitive enough for species or strain-level identification. However, thanks to the unique full-length 16S rRNA sequencing efforts by authors of the Microbial Database for Activated Sludge (MiDAS), who sequenced activated sludge from 750 WWTPs globally, it has become easier to identify core and conditionally rare or abundant taxa on a species-level, and uncover more of their physiological and ecological roles (Dueholm et al., 2021). The same research group is further engaged in another global sequencing effort with focus on sludge from anaerobic digesters.^1^ In agreement with their study, other authors found that the core microbiome consisted of only a few hundred species and is taxonomically composed of very similar organisms (from genus-level upwards) across different reactors globally (Rivière et al., 2009; Kirkegaard et al., 2017).

Analysis by Dueholm et al. (2021) also showed that variation of the microbial community composition of activated sludge (not anaerobic sludge in this case) was shaped by the feed type and immigrating bacteria. They further suggested that many species were functionally redundant because numerous, different amplicon sequence variants (ASVs) were unique across the world, but belonged to the same genera providing similar functions (Dueholm et al., 2021). To provide an overview of the relevant microbial diversity of AD, Table 1 and Supplementary Table S1 provide examples of archaeal and bacterial genera respectively, that were observed in municipal anaerobic digesters globally, including some genus-specific information and their potential role during digestion.

**Table 1:** Examples of common methanogens reportedly present in full-scale anaerobic digesters of wastewater treatment plants and some genus-specific information.

Class	Genus	Temperature optimum	Gram stain	pH _growth_	Motility	Genome size (Mb)^a^	Genes^a^	Substrate usage / Notes	References
*Methanobacteria*	*Methanobrevibacter*	Mesophiles	+	5.0–10	NM	1.7–2.9	1,700–2,020	Hydrogen, carbon dioxide	A, B
*Methanobacteria*	*Methanothermobacter*	Thermophiles	+	5.0–9.0	NM	1.6–1.8	1,750–1,860	Hydrogen, carbon dioxide	A, B
*Methanobacteria*	*Methanobacterium*	Mesophiles	+, −	5.5–9.9	NM	2.4–2.6	2,411–2,584	Hydrogen, carbon dioxide, formate, alcohols or carbon monoxide / filamentous growth	B, C
*Thermococci*	*Candidatus* Methanofastidiosum	Mesophiles	NA	NA	NA	1.5–1.99	NA	Methylated thiol	A, H
*Methanomicrobia*	*Methanolinea*	Mesophiles	−	6.7–8.0	NM	~2.0	~2,108	Hydrogen, carbon dioxide, formate / filamentous growth	D, B
*Methanomicrobia*	*Methanospirillum*	Mesophiles	−	6.5–10	M, NM	~3.5	~3,294	Hydrogen, carbon dioxide, formate, 2-probanol, 2-butanol	A, B
*Methanomicrobia*	*Methanoculleus*	Mesophiles	−	5.0–8.5	M, NM	2.15–2.9	NA	Hydrogen, carbon dioxide, formate / associated with high ammonium levels	A, B, E, F, G
*Methanomicrobia*	*Methanoregula*	Mesophiles	−	4.5–5.5	NM	~2.6	~2,517	Hydrogen, carbon dioxide / acidophilic	B, C
*Methanomicrobia*	*Methanothrix* *^b^*	Mesophiles	−	5.5–8.4	NM	~2.8	~3,008	Acetate / filamentous; often the most abundant genus	A, B, I
*Methanosarcinia*	*Methanosarcina*	Mesophiles & Thermophiles	+, −	5.0–8.9	NM	3.4–5.8	3,434–4,721	Acetate, hydrogen, carbon dioxide, methanol / abundant	A, B

a based on referenced type strains or metagenome assembled genomes.

b Synonym = Methanosaeta, illegitimate name.

References: A (Kirkegaard et al., 2017); B (DeLong et al., 2014); C (Tonanzi et al., 2021); D (Imachi et al., 2008), E (Manzoor et al., 2016), F (Anderson et al., 2009), G (Kougias et al., 2017); H (Nobu et al., 2016); I (Garrity et al., 2011); NM, non-motile; M, motile, Mb, Megabases, NA; not available.

### Diversity as insurance

A common observation is that diversity increases the process resilience and efficacy of AD (Ramirez et al., 2009; Werner et al., 2011). By extending the Anaerobic Digestion Model 1, Ramirez et al. (2009) pointed out that “biodiversity acts as insurance for CSTR functions against temporal changes in environmental factors.” Indeed, it is believed that process stability is directly linked to the functional diversity of the microbial community (Briones and Raskin, 2003), although the mechanisms are not yet elucidated. De Vrieze et al. (2013) evaluated the functional stability of 10 l continuously stirred reactors fed with pulses and suggested that higher variation in substrate composition might promote greater functional stability. Another study, which assessed microbial communities in nine full-scale anaerobic digesters over a year, reported that greater phylogenetic variability and evenness of communities was associated with greater methane production (Werner et al., 2011). The same study also suggested that syntrophic organisms were highly resistant to change, highlighting the need for understanding the role of biomarker metrics for different metabolic groups.

Overall, microbial abundance in anaerobic sludge of WWTPs is typically dominated by bacteria, followed by a relatively small (yet important) population of mostly methanogenic Archaea and an even smaller fraction of eukaryotic organisms. For example, the overall composition in a full-scale mesophilic anaerobic digester in Beijing (WWTP with 1,000,000 m^3^/day inflow) was 93% Bacteria, 5.6% Archaea and 1.1% Eukaryotes (Guo et al., 2015) based on untargeted metagenomic sequencing. Globally, bacteria belonging to the phyla *Proteobacteria, Firmicutes and Bacteroidetes* were commonly found to be among the dominant groups, while *Chloroflexi* and *Actinobacteria* were also present in significant numbers, although this is highly variable (Nelson et al., 2011; Yang et al., 2014; Guo et al., 2015; Kirkegaard et al., 2017).

Among the Archaea, *Euryarchaeota* (according to the conventional and now revised taxonomic classification) was the dominant phylum, usually accounting for 93–100% of archaeal reads. Under mesophilic temperatures *Euryarchaeota* in full-scale reactors are typically dominated by the genus *Methanothrix* (formerly *Methanosaeta*), strictly acetoclastic methanogens, associated with CH_4_ production (Tonanzi et al., 2021). On the other hand, *Methanosarcina* species, which have syntrophic relationships with acetate-oxidising bacteria, dominate under thermophilic temperatures or at high ammonia levels (Karakashev et al., 2006; Ho et al., 2013).

Furthermore, rare taxa, or those with a low abundance when assessed through DNA sequencing, represent the majority of microbial diversity in municipal digesters. While abundant organisms are important indicators of function, rare taxa comprise the large majority of species and provide a critical functional reservoir for process stability and robustness (Lynch and Neufeld, 2015). Rare taxa contribute disproportionately to total metabolic activity during AD (Jia et al., 2019). It is now clear that rare bacteria such as *Thermovirga* in the phylum *Synergistetes* can occupy important niches in the majority of ecosystems and assist in amino-acid turnover (Dahle and Birkeland, 2006). In fact, *Thermovirga* appear to have found a niche in anaerobic digesters of Danish WWTPs and likely play an important role in amino acid fermentation in municipal sewage sludge (Jiang et al., 2021b). Another unexplored knowledge frontier for AD is the role of fungi. Despite their low prevalence it is likely that they fill important functional niches, fundamental to the breakdown of organics during AD (Sun et al., 2015).

Overall, while there are still large knowledge gaps on individual taxa and their function, fundamental ecological concepts or principles apply, including genotypic abundance constraints, niche-or interaction-dependent distributions and functional redundancies, all of which presents an opportunity to predict and manipulate reactor functions. In the following section, studies are presented that examine the effects of changing reactor conditions on microbial ecology and process performance of anaerobic digesters fed with municipal sludge.

### The start-up phase of anaerobic digestion

To study the relationships between the microbial community and their associations to reactor performance it is useful to observe ecological dynamics during controlled, continuous long-term experiments. Our search of the literature for mesophilic digesters (see Supplementary Methods) identified 21 longitudinal studies of continuously stirred reactors, with 10 of these studies investigating microbial diversity using municipal sludge as feedstock (Table 2).

**Table 2:** Selected studies that investigated long-term microbial dynamics during anaerobic digestion of municipal sewage sludge from a search on the Web of Science^™^ database (Clarivate Web of Science Core Collection).

Feed types	Scale	Reactor size	Reactor qty	Temp °C	Days	SRT/HRT	OLR	Aim	Findings	Location	Year
Digested sludge	Full-scale	3,300 m^3^	2	33	315	19	1.75 ^a^	Investigate microbial dynamics during start-up phase	Waste activated sludge was suitable inoculum, clear shifts observed, enrichment of acetoclastic methanogens	Venice, Italy	2021^A^
PS and (T)WAS	Pilot-scale	2 m^3^	4	35	160	51.5, 25.9	1.1, 1.5 ^a^	Effect of phenylacetic acid on performance	Mixed sludge was less affected compared to PS	Santiago, Chile	2015^B^
PS/WAS mix	Lab-scale	2 l	various	37	93	20	3.7–9.4 ^b^	Study effect of mixing	Increased mixing inhibited syntrophic oxidation of VFAs	Urbana, USA	2001^C^
PS	Lab-scale	4 l	6	36	42	-	3.4–5 ^b^	Study effect of OLR	Increased loading promoted methane production; the community was unaffected	Wooster, USA	2011^D^
PS/WAS mix	Lab-scale	0.5 l	6	55	319	5–30	1–2 ^b^	Study effect of biochar	Cornstove biochar increased methane content and flow rate	Lemont, USA	2017^E^
PS/WAS mix	Lab-scale	3_w_ L	6	35	130	15	3 ^b^	Study effect of antibiotics	Reduced methane production, increased risk of AMR	Changsha, China	2019^F^
(T)WAS	Lab-scale	1.5_w_ L	4	37	155	15	2.7 ^c^	Study effect of synthetic musk	Synthetic musk inhibited hydrolysis	Sydney, Australia	2020^G^
Food/WAS mix	Lab-scale	10 l	2	36	100	20	0.8–1.7 ^b^	Understanding instability	Mixing WAS with Foodwaste increased stability, related to trace elements and microbes	Monterotondo, Italy	2020^H^
(T)WAS	Lab-scale	1.3 l	4	35	300	12	1.4 ^a^	Study effect of nitrous acid pre-treatment	Free nitrous acid pre-treatment improved methane yields but also increased pathogens	St Lucia, Australia	2021^I^
(T)WAS	Lab-scale	0.5 l	2	35	60	5	-	Study effect of contaminants on stability	Cell lysis by plastic additive, but process was stable	Changsha, China	2021^J^

Refer to Supplementary materials for search criteria; Reactor size = total volume unless marked `w` for working volume.

a kgVS m^3^ day^-2^.

b gVS L^-1^ day^-2^.

c gCOD L^-1^ day^-2^; SRT, sludge retention time; HRT, hydraulic retention time; OLR, organic loading rate; PS, primary sludge; WAS, waste activated sludge; TWAS, thickened waste activated sludge. References: A (Tonanzi et al., 2021); B (Cabrol et al., 2015); C (McMahon et al., 2001); D (Gómez et al., 2011); E (Shen et al., 2017); F (Xu et al., 2019); G (Wei et al., 2020); H (Tonanzi et al., 2020); I (Calderon et al., 2021); J (Tang et al., 2021).

One of these studies observed microbial dynamics during the start-up phase of AD. Tonanzi et al. (2021) filled and fed a municipal digester tank (3,300 m^3^; 19-day hydraulic retention time (HRT)) with waste activated sludge (WAS; ~5% total suspended solids, organic loading rate 1.75 kg_VS_ m^3^ day^−1^) to monitor taxonomic changes over a period of 315 days at a mean temperature of 33°C (Table 2). The study illustrated how the microbial population shifted from an aerobic to anaerobic hydrolytic and fermentative metabolism. They found that microbial growth and methane production stabilised only after 100 days (>5 HRTs). During the first HRT no methane was produced and negligible amounts of volatile fatty acids (VFA) were utilised. This coincided with a temporal collapse of bacterial and archaeal populations, indicating a complete population shift, during which the microbiota of the WAS adjusted to the anaerobic conditions in the tank. The authors further showed that total archaeal growth in the digester tank increased and stabilised to a higher level compared to the start-up period, which coincided with increases in methane production, volatile fatty acid degradation and pH increases to around 7.

### Long-term dynamics and responses

Microbial composition and abundances remain variable even after the start-up phase. For example, Wu et al. (2016) fed triplicate digesters with dairy waste for 2 years and detected a clear successional pattern with ongoing and increasing niche differentiation. For Tonanzi et al. (2021) microbial composition and abundances kept changing even after 200 days of operation, despite otherwise consistent WAS quality and stabilised levels of total bacterial/archaeal biomass, VFA degradation and methane production.

Furthermore, changes in loading rate were assessed in a bench-scale control experiment using 4 l reactors fed with sludge from a full-scale reactor (Ohio State, United States), which was diluted with water to adjust loading rates from 3.4 to 5 g*_vs_* L^−1^ day^−1^ (increasing loading rate by nearly 50%). The authors found that methane production increased without affecting the composition of some specific archaea (Gómez et al., 2011; Table 2). Other prokaryotes were not investigated in this study. However, it pointed to an archaeal community that was resistant to change.

Control experiments also highlight the effects of toxins on long-term dynamics of AD. For example, phenylacetic acid is a toxic phenolic degradation product of detergents and surfactants found in personal care items. In a controlled experiment, a single pulse of phenylacetic acid was added to primary sludge sampled from a full-scale WWTP (Santiago, Chile), which was fed to a total of four pilot-scale (2 m^3^ each) continuously stirred reactors (Cabrol et al., 2015). In digesters fed only with the primary sludge, phenylacetic acid caused VFA accumulation, reduced biogas production and a clear reduction in the dominant acetoclastic *Methanothrix* sp. (formerly *Methanosaeta*), while the hydrogenotrophic *Methanospirillum sp*. remained relatively stable.

However, in the reactors fed with a mix of primary and secondary sludge (60/40%), the archaeal community was completely unaffected by phenylacetic acid during the 160 days of operation. The authors speculated whether the increased stability in the mixed sludge reactors was due to more broad hydrogen utilisation from the fermentative and oxidative diversity (due to a more mixed/diverse feedstock), which may have ensured low partial pressure of hydrogen (p_H2_) and kept the thermodynamics favourable for methanogens. Hence, this study again highlighted the importance of the metabolic reservoir of microbial diversity for process stability.

Another long-term study by Calderon et al. (2021) compared the performances of four 1.3 l mesophilic and stirred digesters after the TWAS-feed was pretreated with free nitrous acid (FNA), a known inhibitor of obligate anaerobic microbes. It showed that abundances of *Methanothrix* spp. remained stable or increased compared to the untreated control. It seemed the methanogens were resilient, and possibly increased in response to increased VFA and hydrogen flux (induced by FNA treatment). However, increased methane yield was accompanied by the growth of opportunistic pathogens, with pathogens able to utilise organic carbon solubilised by FNA pre-treatment.

Taken together, these studies highlight the need to understand the ecological mechanisms that predict the observed phenomena after changing conditions during AD. However, most of these current studies do not look at broad shifts in functional clades, particularly for front-end organisms, which presents the greatest diversity in AD. Exploring the knowledge gaps opens opportunities for improved reactor management in future for operators of WWTPs.

## The path ahead for integrating ecological markers

The foundational principle of ecology is that everything is connected, and this principle will drive the choice of tools used to develop and integrate ecological markers in biological systems. Investigations will inevitably require the handling of multiple, large datasets and find process-relevant associations. Improving digester function through the application and integration of advanced analytical tools will therefore involve a new generation of bioinformaticians and data scientists. In the following section some of the benefits of this approach, as well as perspectives on methods are discussed.

### Benefits of using ecological markers for operators

The potential practical applications arising from investigations into sludge ecology are extensive. Ecological exploration will assist in the development of risk-management strategies or optimising operational endpoints. The commonly used physicochemical reactor metrics, such as pH, alkalinity, volatile solids destruction, gas flow rate, or ammonia and acetate accumulation are endpoints of the combined function of the microbiome. These metrics manage the process, not the microbiome, hence they do not inform about underlying community function or resilience and do not prevent problems, such as bulking, foaming, and low biogas production from occurring. It is therefore necessary to integrate such process derived data with data attained from DNA/RNA, proteins and/or metabolites and map them to the process.

Research outcomes may include new management options to increase stability, reduce foaming, monitor pollutant risks or to optimise COD conversion to methane. For example, if we understand how changes in carbon sources in mesophilic reactors are associated with metabolic traits, and particularly hydrolytic capacity, it will assist in the development of stimulants to optimise community composition for the degradation of volatile solids. The metabolic drivers for variable exocellular hydrolytic performance are not understood and could be related to microbial prioritisation (expression) and thermodynamic responses. This knowledge may then lead to amendments to promote certain energy pathways and improve hydrolysis.

Other potential applications include the indirect monitoring of pollutant loads by proxy of shifts in diversity. One example are *Dehalococcoides* spp., which are biomarkers for organohalide compounds in wastewater (Lu et al., 2017). Similarly, sludge-ecology can assist in the application of beneficial microorganisms and improve their survival once applied to the reactors. ‘Phage therapy’ emerged as a potential strategy to control filamentous growth or pathogens (Withey et al., 2005; Petrovski and Seviour, 2018), although it has not been applied to the full scale yet. Furthermore, the applications of the recently discovered ‘filament-eating’ bacteria, could be implemented to remove a range of foam-associated filamentous bacteria from sludge (Batinovic et al., 2021).

Microbial biomarkers or risk scores, could indicate the need for prevention (Carballa et al., 2015). Biomedical research already considers the gut microbiome a clinical feature, whereby the rate of compositional change after treatment is associated to clinical outcomes, with higher rate of change being indicative of type 1 diabetes, for example (Gilbert et al., 2018). Consequently, with ongoing research, biome-based monitoring of processes, pathogens, and pollutants will become a commercial reality for WWTPs within this decade.

#### Prevention of biological foaming

Prevention or elimination of stable biological foaming during AD (and during processing of WAS feed) is arguably the biggest opportunity for increasing operational efficiency as it is a widespread and pervasive problem globally, requiring ongoing management (Pagilla et al., 1997; Foot and Robinson, 2003; Moeller and Görsch, 2015).

Foaming mechanisms and management have been reviewed extensively and involve (bio)surfactants, hydrophobic matter and rising gas bubbles (Foot and Robinson, 2003; Ganidi et al., 2009; Dalmau et al., 2010; Petrovski et al., 2011; Yang et al., 2021). Factors that increase microbial foam potentials are commonly associated with a change in the sludge composition or environmental conditions, such as temperature changes, changes in mixing regimes, higher organic loading rates or a high percentage of activated sludge feed that may lead to increases in VFA and ammonia concentrations (Pagilla et al., 1997; Foot and Robinson, 2003; Moeller et al., 2012; He et al., 2017). However, it is unclear to what extent microbial ecology processes increase foaming potentials through the production of biosurfactants and hydrophobic metabolites.

Importantly, microbial cells may affect foaming potentials even after cell death through the release of hydrophobic materials (Petrovski et al., 2011), which has implications for the accuracy of genomic methods used for assessing foaming potentials. Foaming can be associated with the presence of potentially dead or non-growing, hydrophobic, filamentous bacteria that enter anaerobic digesters from WAS (Foot and Robinson, 2003). From microscopy and genomic sequencing it is well established that several filamentous genera (including ‘*Candidatus* Microthrix spp.’) and mycolic acid producing bacteria (e.g., *Gordonia spp*.) are agents for bulking and foaming in activated sludge and anaerobic digesters (Dillner Westlund et al., 1998; De Los Reyes and Raskin, 2002; Rossetti et al., 2005; Kragelund et al., 2007; Nierychlo et al., 2020b). These organisms therefore represent taxonomic markers that indicate foaming potentials, albeit not reliably yet.

The further development of relevant biomarkers may enable early intervention by operators. Future research should explore foaming biomarkers from controlled long-term experiments in lab-scale reactors using feedstock from full-scale plants. Methods to establish foam potential tests (e.g., Blackall et al., 1991; Lunkenheimer et al., 2010; Fryer et al., 2011; Jiang et al., 2018) require international harmonisation to enable robust assessment of links of microbial communities dynamics with foam potentials. Moreover, increases in sludge surfactant activity and cell-surface hydrophobicity (Rosenberg, 2006; Chao et al., 2014) may be associated with clades of organisms that bloom in a changing sludge environment, hence such sludge measurements are essential for metagenomic foam assessments.

#### Increasing biogas recovery in mesophilic reactors

Biogas produced during AD consists mainly of methane (CH_4_, 40–75%) and carbon dioxide (CO_2_, 25–60%), plus some small amounts of hydrogen (H_2_), nitrogen (N_2_), ammonia (NH_3_), hydrogen sulfide (H_2_S) and water (H_2_O). The conversion efficiency of organic materials in sludge into valuable methane is directly linked to the sludge composition, whereby primary sludge consists of more biodegradable materials and produces more VFAs compared to WAS (Araujo et al., 1998; Ucisik and Henze, 2008). Around 53 to 65% of the volatile solids of primary sludge and 30 to 50% of the volatile solids of WAS are biodegradable without prior solubilising treatments (Parkin and Owen, 1986; Araujo et al., 1998). Surprisingly little is known about the relevant microbial dynamics and whether WAS degradability can be enhanced through careful management of the microbiome.

However, one of the greatest research-interests in AD relates to pre-treatment and co-digestion of sewage sludge to release biodegradable carbon materials, thereby increasing the rate of hydrolysis and ultimately energy efficiency of mesophilic digesters (Mata-Alvarez et al., 2014). For example, AD of thermally pre-treated (THP) WAS can double methane production compared to regular WAS, particularly at long WAS SRTs (Batstone et al., 2009). This has been accompanied by a switch from acetoclastic to hydrogenotrophic methanogenesis, likely due to H_2_ partial pressure as well as increased ammonia concentrations (Zhang et al., 2021). Furthermore, anaerobic co-digestion with other types of organic wastes, such as municipal organic wastes or fat, oil and grease or dairy wastes can improve the economic feasibility of WWTPs, mainly due to the benefits of increased gate fees, energy neutrality from biogas recovery or pollution dilution (Mata-Alvarez et al., 2014; Sembera et al., 2019).

Nonetheless, depending on the type of organic waste used for co-digestion, problems have shown to arise from the accumulation of solids, VFAs or pollutants, as well as from high backloads of nitrogen, reduced dewaterability or increases in hydrogen sulfide production (Mata-Alvarez et al., 2014; Sembera et al., 2019). Hence, reviewers on this topic emphasised the need to be able to better model microbial dynamics to predict co-digestion processes and to be able to choose the best co-substrate and blend ratios (Mata-Alvarez et al., 2014). To progress this field, controlled long-term studies are required that show microbial dynamics over time to gain confidence about the stability of the processes under various scenarios.

#### Improving risk management of pathogens and pollutants

Risk-based management for biosolids-reuse involves the interruption of disease transmission pathways by inactivating pathogenic biological agents or vectors, but also the monitoring of organic and inorganic pollutants (Ahring, 2003). In the medium to long-term, novel on-site sequencing technologies, coupled with empirically derived database biomarkers has the potential to monitor and manage surges of a wide range of different pathogens, antibiotic resistance risks, or complex mixes of toxic elements in sludge.

The concept of using DNA for environmental monitoring is not new and was proposed to become the next-generation monitoring tool for a wide range of aquatic ecosystems (Valentini et al., 2016). While indicator organisms are commonly assessed to manage pathogen-loads in WWTPs, we do not yet fully understand causal relationships between pathogens and other organisms in sludge communities. Some known examples include organisms such as *Campylobacter jejuni,* which does not compete for resources as it exclusively uses specific amino acids and vitamins, or bacterial pathogens, such as *Salmonella* spp., *Yersinia spp*., *Listeria spp*., and *E. coli*, which compete with other bacteria for carbohydrates (Zhao and Liu, 2019). Currently, this type of knowledge is not exploited for making precision diagnoses for AD, while is already practiced in biomedical research for example (Gilbert et al., 2018).

Furthermore, toxic pollutants trigger community responses that can be captured as distinct compositional changes over time. For example, compositional differences in multiple full-scale mesophilic anaerobic digesters in the Czech Republic were indicative of the presence of metals and some specific organic pollutants such as the flame retardant hexabromocyclododecane (Stiborova et al., 2020). In addition, some specialised organisms may indeed grow on and transform some organic pollutants, making their taxonomic abundance a proxy for pollutant load of specific hydrocarbons. One of the most widely known anaerobic indicator organisms are *Dehalococcoides.* The presence of *Dehalococcoides spp*. clearly indicated the presence of organohalides in sludge and sediments (Lu et al., 2017). Moreover, microplastics can result in significant functional changes to the digester biome. Polyvinyl microplastic promotes a dramatic decrease in fermenting bacterial species, such as *Proteiniborus* sp., *Bacteroides sp*. and *Rhodobacter sp*., and also the methanogenic *Methanothrix sp*. (Wei et al., 2019). Exposure to polystyrene microparticles causes a decline in the relative abundance of several bacterial families, such as *Gracilibacteraceae* and *Anaerolinaceae,* whereas an increase in abundance of other bacterial families, such as *Geobacteraceae* and *Desulfobulbaceae* was reported (Fu et al., 2018).

Nonetheless, the potential for microbial communities to become risk-markers may depend on the type of pollutant, their bioavailability and if it is present in great enough concentrations to provide energy for microbial growth or lethality to inhibit growth (Beek, 2006). Some of the most difficult-to-degrade organic pollutants, such as highly halogenated organohalides or perfluoroalkyl and polyfluoroalkyl substances (PFAS) provide little energy for growth, and it remains doubtful that microbial communities will become useful markers for such important xenobiotics. Although in pure culture a few organisms have indeed been shown to dechlorinate and defluorinate organic compounds, further research will become critical to understand how these organisms can be useful in mixed communities during AD with the aim to increase the repertoire of indicator organisms to supplement risk assessment strategies in future (Beek, 2006; Huang and Jaffé, 2019).

### Biomarker discovery through targeted or untargeted sequencing

For operators of anaerobic digesters, predicting desired process endpoints or risk factors from community interactions is a practical outcome from this type of research. Microbial community compositions and interactions are early ‘signals’ that predict successional trajectories of microbiome function. Hence, different biomarkers can be useful for digester monitoring and are potential parameters for more complex predictive models. A biomarker is a biologically derived metric that is correlated with a process or risk of interest and can include diversity indices, as well as the presence, abundances and flux of genes, microbial groups, or metabolites. Some diversity indices are discussed in more detail below. Data exploration and method development is a research-priority that will enable the application of biomarkers for predicting processes and risk factors in wastewater treatment trains.

Two main sequencing approaches are available to generate data for biomarker discovery: targeted (amplicons) and untargeted sequencing (shotgun or long-reads). Although, targeted (amplicon) sequencing on its own is likely insufficient to produce robust biomarkers (De Vrieze et al., 2018), it is currently the most economical method to assess prokaryotic diversity. Amplicon sequencing assesses diversity based on a conserved region of the 16S rRNA gene in prokaryotes or the ITS region in fungi. The resulting amplicon sequences (or amplicon sequence variants) can also be used to indirectly predict enzyme abundances with packages such as PICRUSt2 (Phylogenetic Investigation of Communities by Reconstruction of Unobserved States, Douglas et al., 2020). While there are important limitations to PICRUSt2, including its dependence on an appropriate reference genome,^2^ it has seen significant improvements in predictions of metagenome functions since the first version was released in 2013. For example, compared to other functional prediction tools, PICRUSt2 predictions of KEGG Ortholog (KO) abundances has some of the highest correlations with shotgun metagenomic sequencing (Douglas et al., 2020). Such functional prediction tools can be useful for investigators, but we highlight that its default reference genome requires further validation for engineered AD environments.

While predicting functional biomarkers is difficult even with shotgun and long-read metagenomic approaches, one of the advantages of targeted prediction tools is the ability to predict and compare phenotypes (e.g., the presence of specific carbon utilisers or denitrifiers) without background noise (non-target DNA) and without excessive per-sample costs. However, it is important to understand that a phenotype or functional potential is not evidence for expression of that phenotype or function. Nonetheless, such data can be explored to find predictors or biomarkers for a desired process during AD.

A limitation of targeted sequencing approaches are the various sources of abundance biases. Tools such as PICRUSt2 attempt to normalise gene abundances to copy numbers of 16S rRNA genes present in different organisms. This can minimise any abundance bias for species with higher numbers of 16S rRNA genes. However, it was noted that these normalisation methods can be highly variable (Louca et al., 2018) and do not account for polyploidy, which is another source of abundance bias (Soppa, 2017). Indeed, there is a fundamental dearth of basic information on ploidy within the prokaryotes, and given that, should prompt practitioners to not ‘over interpret’ small differences, to treat the data as ordinal (*cf.,* quantitative), and to encourage the use of multiple lines of evidence. Other limitations of amplicon sequencing and subsequent functional predictions are uncertainties due to bias of primer and database, and the reliance on known taxa or enzyme-encoding genes. However, thanks to a highly engaged global research community these databases are rapidly improving.

In comparison, non-targeted metagenomic sequencing approaches (shotgun or long-read sequencing) capture all known and unknown enzyme-encoding genes across all three domains (eukaryotes, bacteria and archaea). However, the required sequencing depths (~10–20 gigabases) per sample for non-targeted metagenomic sequencing is prohibitive (De Filippo et al., 2012). These approaches require increasingly specialised bioinformatic expertise to assemble, bin and annotate sequences (Sieber et al., 2018). However, new packages, such as SqueezeMeta, are appearing that offer fully automated bioinformatic analysis pipelines (Tamames and Puente-Sánchez, 2019).

A major advantage of non-targeted metagenomic sequencing is the ability to assemble genomes of individual organisms. Metagenome-assembled genomes (MAGs) help determine the functional potential of assembled genomes, including those of unknown bacteria and archaea (Singleton et al., 2021). High-throughput long-read sequencing technologies, including Oxford Nanopore platforms, have made such assembly easier. Singleton et al. (2021) demonstrated how the combination of long-and short-read sequencing methods enabled the assembly of 1,083 (near-) complete genomes, which included the full-length 16S rRNA genes and 23S and 5S genes, across 23 WWTPs in Denmark. From the identifed functions (nitrification, denitrification etc.) the authors highlighted that a large undescribed diversity exists in these engineered systems even within well-studied functional guilds. Metagenome-assembled genomes discovery will be valuable to improve databases for future targeted studies as it is possible to map amplicons and abundances from targeted sequencing to the full functional potential of MAGs.

**Figure 2:**
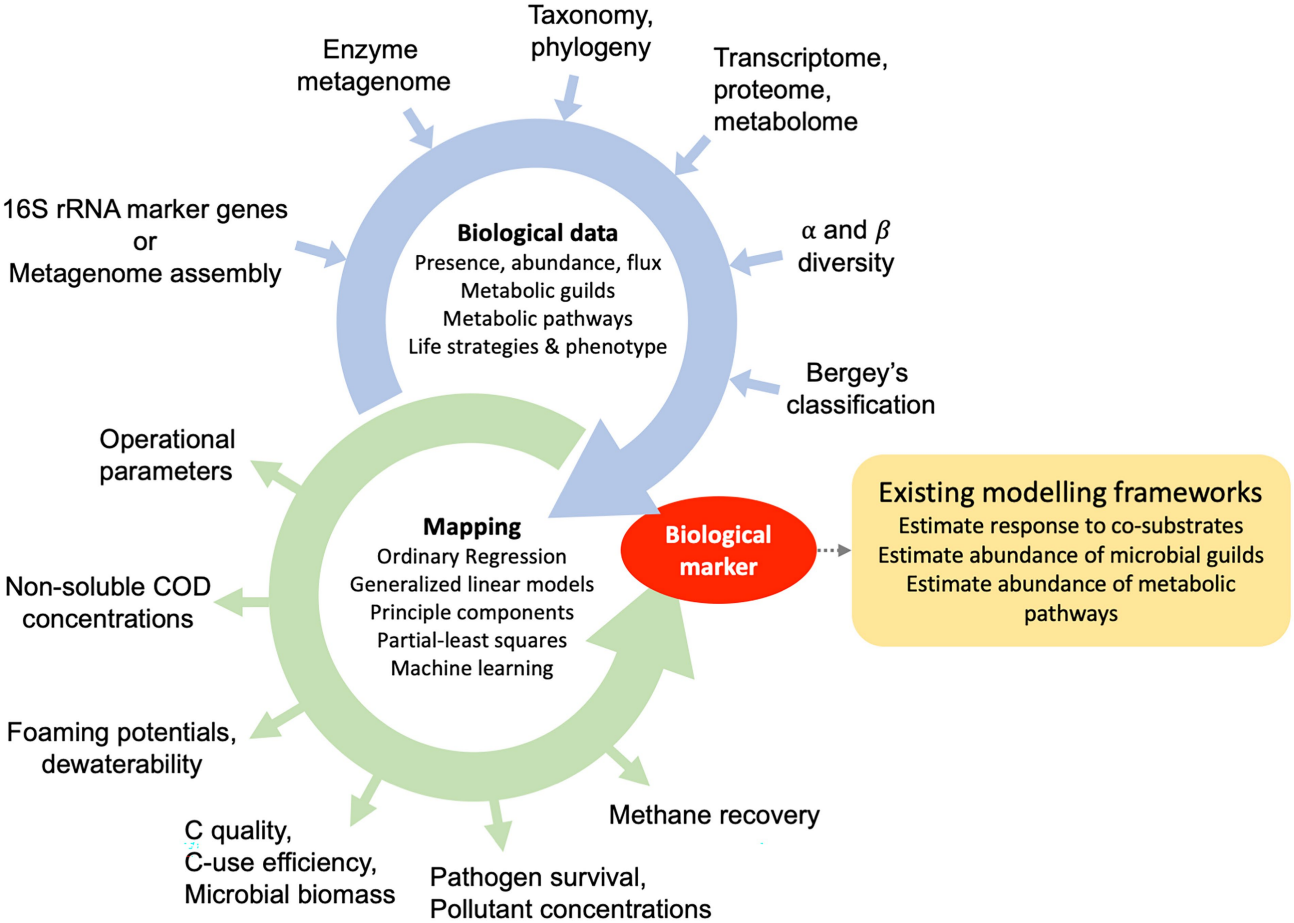
Pathways for the development of process-biomarkers during anaerobic digestion of municipal sewage sludge. Traits, phenotypes and life strategies are quantified using culture-dependent and independent data sources, which can include the presence, abundance or flux of metabolic guilds (microbial groups), metabolic potentials, the metabolic profiles or life-strategy classification schemas.

Once sludge DNA is sequenced, processed, and fully assessed, relevant microbial taxa or traits and their abundances can be concatenated into a dataset together with other reactor metrics to create predictive models (Figure 2). There are a large number of regression or machine learning methods to predict and infer biomarkers and were reviewed for studies on the human microbiome (Marcos-Zambrano et al., 2021). For this purpose, it is important to consider that any abundance data, derived either from targeted or non-targeted sequencing approaches, is compositional (Gloor et al., 2017). In other words, increasing abundances of one group of microbes or enzymes can be the result of other microbes/enzymes decreasing. The inherent qualities of compositional data has led to the use of log-ratios to develop biomarkers from microbiome data and is further described elsewhere (Pawlowsky-Glahn and Egozcue, 2006; Filzmoser et al., 2018). For example, a new ratio-based algorithm was recently proposed by Gordon-Rodriguez et al. (2021) and was made available with the R package CoDaCoRe.^3^

A biome-informed biomarker is a process-related metric and once validated, can be used to diagnose relevant sludge processes. However, it is possible to further integrate biomarkers into existing metabolic modelling frameworks by adjusting parameters. Approaches for metabolic modelling of mixed anaerobic systems, including their limitations, were reviewed elsewhere (Batstone et al., 2019). Broadly, two approaches for metabolic modelling are applied. They are (1) cellular-level and (2) biochemical-process modelling. Novel approaches were highlighted, such as stoichiometric metabolic networks for multispecies cellular-level models (Perez-Garcia et al., 2016) or individual-based bioprocess modelling (Batstone et al., 2006). These models can be used to generate mechanistic hypotheses and improve process efficiency through control and optimisation and may be driven by the desire to improve a particular process during AD. Overall, biome-derived markers have the potential to optimise parameters of existing models.

#### Integration of meta-omics

**Figure 3:**
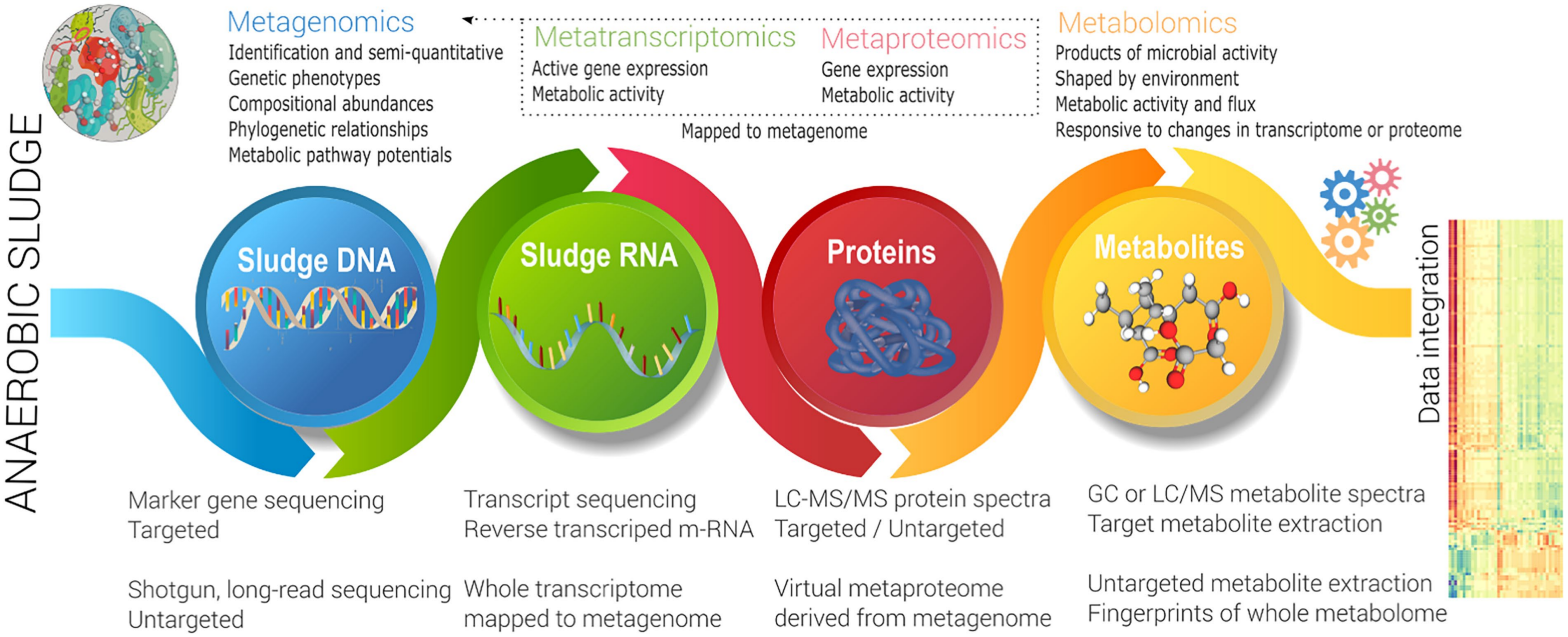
Schema of a meta-omics approach.

As noted in the previous section, the focus to date has been DNA-centric biomarkers, either differentiating whole communities utilising broad metrics, or relatively low resolution (semi-quantitative) biomarkers for characteristic (generally undesired) function such as foaming or inhibition response. This does not address the problem of investigating and enhancing front-end function, which as noted earlier is key to broad improvement in overall system performance. Also as noted above, a lack of knowledge of specific microbial capability, as well as functional redundancy challenges this approach. This likely requires the use of activity (rather than phylogenetic) focused techniques to link function back to identity. This can be targeted (to specific microbes, enzymes or metabolites), and has been very successfully used in the back-end where actors are known (see Section 2), but a meta-omics approach (Figure 3) is more effective for discovery of unknown information (Vanwonterghem et al., 2014b). Vanwonterghem et al. (2014b) defines how meta-omics approaches functional potential, expression, and activity of a whole community. The *metagenome* provides the complete enzymatic potential of a community, *via* representative whole (or near whole) individual (often replicated) genomes of representative members.

The *metatranscriptome* also requires nucleic acid sequencing, but sequences reverse transcribe m-RNA in an active community to identify level of expression, and hence metabolically active organisms (Mutz et al., 2013). The *metaproteome* identifies functional enzymes (and other proteins), utilising LC–MS/MS to identify protein mass spectra at a specific time (Langley et al., 2013). Salvato et al. (2021) noted that public protein reference databases may not have close analogous organisms, may have missing sequence information, and are hence less reliable than a database that is directly derived from a metagenomic analysis of the same samples or environment. Metatranscriptomics also benefits from related metagenomics data (rather than public references) to contextualise gene reads and provide differential expression. Metatranscriptomics and metaproteomics both identify expression of either gene or protein respectively, noting there may be differences between the two (Salvato et al., 2021). Both techniques are differential, in that that replicated sampling including a control (baseline) must be done (Vanwonterghem et al., 2014b). Both techniques have advantages and disadvantages, with several drivers (lower per sample cost, improved analytical techniques, easier reconstruction of a virtual metaproteome, direct analysis of proteome, better extraction techniques, use in pure culture biotechnology) favouring metaproteomic analysis.

The metabolome involves extraction of metabolites (involved in primary and secondary metabolism) from environmental samples (Jones et al., 2013), and hence directly represents activity (rather than capability or expression). Metabolomics directly identifies underlying biochemical activities, allowing metabolic pathway reconstruction. For a ‘biological system’ comprising a taxonomically diverse microbiome (as in sludge), the pool of metabolites is diverse in origin, physical and chemical properties, belonging to any variety of classes, such as amino acids, lipids, mono-, di-and tri-saccharides, sugar phosphates, sugar alcohols, organic acids and free fatty acids representing a comprehensive and wide range of concentrations. The ‘metabolome’ therefore directly reflects the metabolic fluxes of a population in anaerobic digesters (Figure 3). It is a DNA independent technique, allowing metabolic reconstruction, but does not directly identify the microbe responsible.

As with genome focused techniques, the application of metabolomics can be targeted (i.e., investigating fluxes of specific metabolites) or untargeted (i.e., comprehensive - investigating all possible metabolites), whereby sample preparation and analytical platforms are more complex for untargeted approaches (Beale et al., 2018). Gas chromatography (GC) or liquid chromatography (LC) coupled with mass spectrometry (MS) are the most commonly used analytical platforms for detecting and measuring, both semi-quantitatively and quantitatively, metabolites with methods previously reviewed (Dettmer et al., 2007; Beale et al., 2018).

#### Alpha diversity

To represent sample diversity, alpha diversity indices can be calculated from abundance tables of ASVs, MAGs, microbial groups, as well as enzyme and pathway potentials. Alpha diversity is suspected to have varying levels of importance for different clades, being more important for fermenters (given variation in substrate types) and perhaps less important for niche specialists such as aceticlastic organisms. Different alpha diversity are applied to ecological studies but all reflect some form of the effective number of individuals for each sample, without considering phylogenetic relationships between individuals (Leinster and Cobbold, 2012). For example, Carballa et al. (2015) proposed that decreasing values of evenness of bacteria or individuals in the genus *Methanothrix* (formerly *Methanosaeta*) could be a warning signal for unstable reactor performance. The evenness index reflects the distribution of individuals, with high values indicating an even distribution of the abundances of individual organisms, which in turn indicates a greater diversity of metabolic activity (Hill, 1973).

#### Beta and phylogenetic diversity

However, the use of biomarkers, based on simple alpha diversity indices alone, may not be sufficient. Beta diversity indices, which are values that define how the diversity of a sample relates to other samples, can further help to indicate stress factors. High values of beta diversity over time indicates a high species-turnover and can be associated with stress, as concluded for copper-contaminated soils by the Microbiome Stress project (Rocca et al., 2019). Beta diversity is often presented along two axes of an ordination of non-metric dimensional scaling (NMDS) using dissimilarity indices such as Bray Curtis, for example, and is further described elsewhere (Borcard et al., 2011). It is an intuitive depiction of sample diversity, because sample points closest to each other have the most similar community composition. However, these common alpha or beta diversity indices ignore phylogenetic relationships, which may be critical if biomarkers are needed to highlight problems in digesters that are caused by phylogenetically interdependent groups of bacteria or archaea.

**Figure 4:**
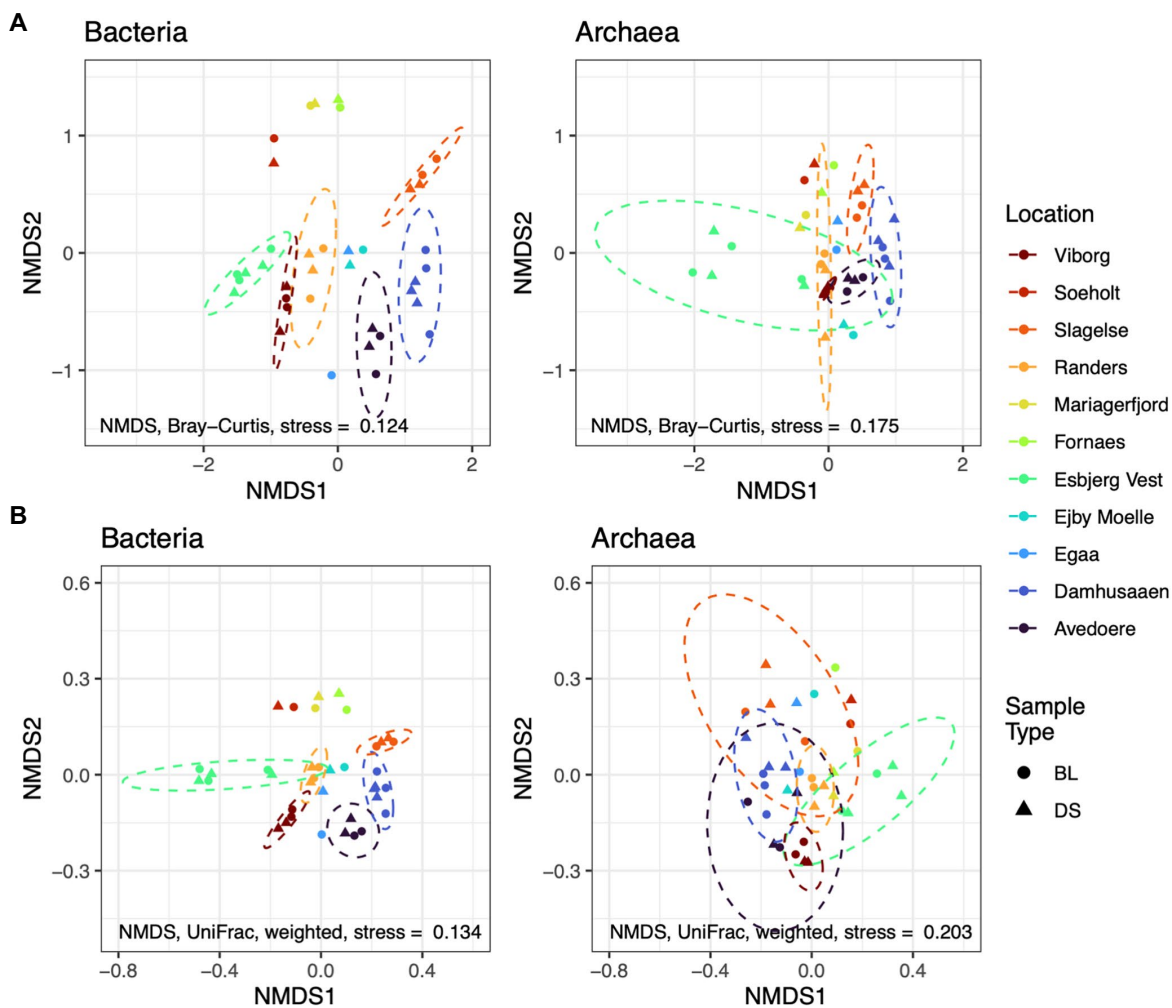
Ordinations of non-metric dimensional scaling (NMDS) using Bray-Curtis distances **(A)** compared with UniFrac distances **(B)**; based on re-processed data from Jiang et al. (2021a) accession number PRJNA645373. Abundances of amplicon sequence variants were filtered to a minimum of 5 reads prior to analysis. Dashed circles show the t-distribution at the 0.95 level in locations with >2 samples. Sludge of digesters from different Danish locations underwent a foaming potential test, whereby the authors sampled the bubble layer (BL) and the digester sludge (DS). No differences in phylogenetic diversity are apparent between BL and DS (not considering abundances, i.e., unweighted). Instead, phylogenetic diversity appears to differ between locations, especially for Bacteria. Information on data processing is available in Supplementary materials.

Alternatively, new alpha and beta diversity indices have been developed that include a measure of the distance between organisms in a phylogenetic tree (Lozupone and Knight, 2005; Faith et al., 2009). This allows the diversity-metrics to be representative of genetic differences, as can be determined from phylogenetic relationships, based on 16S rRNA amplicon profiles. To provide an example of a phylogenetic diversity metric (here the weighted UniFrac distances), and to compare it with the conventional Bray-Curtis distances, we have re-processed public amplicon sequences (accession number PRJNA645373). The data was provided by a research team that conducted an experiment on Danish digester sludges (from various WWTPs), which aimed at identifying foam-associated organisms (Jiang et al., 2021a; Figure 4). See Supplementary Materials for more details on data and processing.

The ordinations show differences in microbial composition between the digester sludges from different treatment plants; the composition of Archaea was more similar between the different WWTPs, compared to Bacteria. However, when phylogenetic information is included into the metric (e.g., using the weighted UniFrac distance), the same sample-compositions appears to be more similar. This suggests that phylogenetic indicators can account for some genetic redundancy in the system. The abundance of an organism in one digester might be different, compared to another digester. However, the abundance and function of that organism might be replaced by a phylogenetically related organism in another digester. Hence, UniFrac distances are more robust biomarkers for identifying biologically important differences in diversity. Further research should test the utility of phylogenetic biomarkers for identifying stress and performance risks.

**Figure 5:**
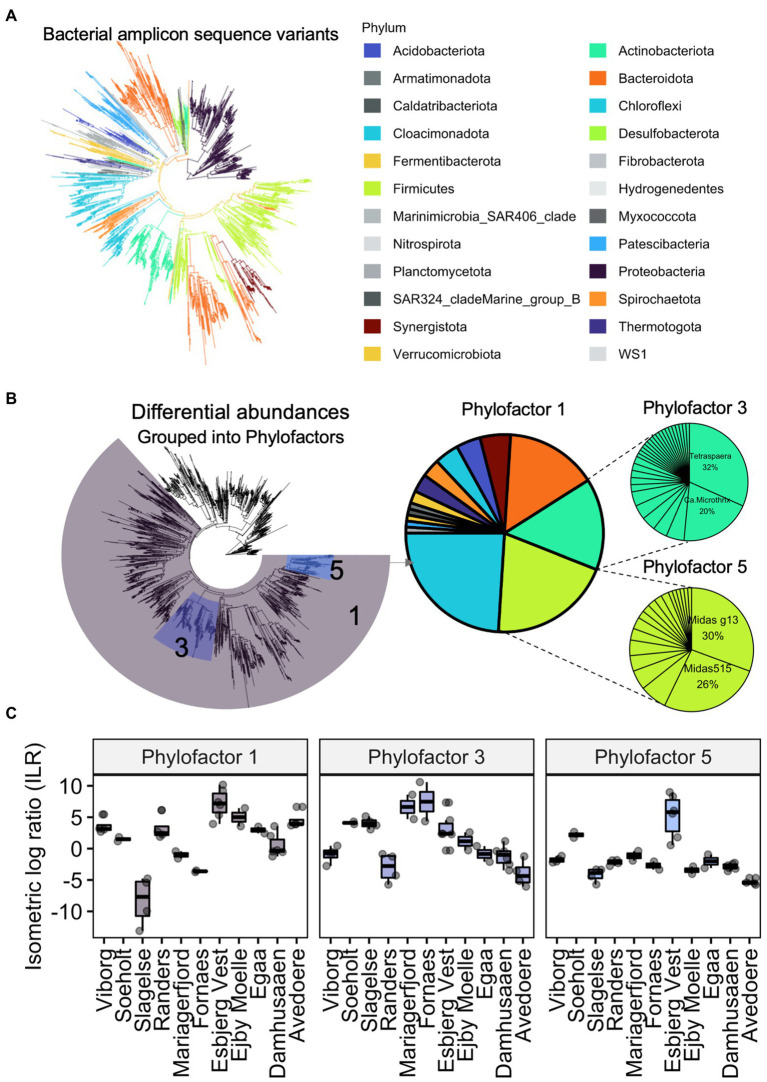
Example of a Phylofactor approach using data from Jiang et al. (2021a). Bacterial amplicon sequence variants and their phylogenetic relationships are represented as a phylogenetic tree **(A)**. Abundances in the eleven Danish digesters were different to each other. Abundances of three groups of bacterial ASVs (phylofactor 1, 3, 5), transformed into isometric log ratios, were relatively different to the remaining bacteria. The relative abundances of bacteria within each of the three phylofactors are showed in pie charts **(B)**. Boxplots show how much the abundances differed relative to each digester **(C)**. For example, relative abundances of all *Actinobacteriota* (Phylofactor 3), composed mainly of the genera *Tetrasphaera* and *Ca.* Microthrix, were different in the digesters.

Phylogenetic differences in digester sludge can be further characterised according to changes in the relative abundance of specific phylogenetic groups. For example, the package phylofactor helps to identify patterns of changing relative abundances among samples (Washburne et al., 2019). Instead of comparing taxonomy abundances on a specific taxonomic level (e.g., phylum or genus), phylofactorisation finds and highlights changing abundances among groups of varying levels of taxa, along a phylogenetic tree. Using the same data set from Figure 4, we performed phylofactorisation on the bacterial community after creating a phylogenetic tree (Figure 5). It highlights how abundances of bacteria, including the clade of *Actinobacteriota* varied in digester sludge from different Danish locations. It further showed that two filamentous genera, *Tetrasphaera* and Candidatus *Microthrix*, comprised over half of these varying abundances in the phylum *Actinobacteriota,* which also included other filamentous organisms such as *Gordonia* spp. Supplementary Table S1 presents more information on *Tetrasphaera* and *Ca.* Microthrix. In future, this type of analysis will help to provide phylogenetic ‘signals’ of changes in reactor performance over time. There are a range of other approaches to quantify differences in abundances of microbial groups, including ALDEx and ANCOM, which were recently reviewed elsewhere (Lin and Peddada, 2020; Nearing et al., 2022).

#### Trait-based phenotypes

In addition, ecological microbiome studies have started to explore the use of community-level functional traits to predict ecosystem function (Martiny et al., 2013; Wood and Franks, 2018; Malik et al., 2020). Functional traits are potentially more robust predictors of a sludge phenotype than taxonomic composition because metabolic capabilities of individual species can in fact be different among related taxa (Wood et al., 2015). Generally, trait-bases assessments assume that microbial communities switch between different metabolic strategies, constantly trading off their physiological abilities and evolutionary opportunities (Xu et al., 2014). Trait-based ecology attempts to group microbial communities according to their life strategies that are based on the genetic potentials from enzyme profiles and their inferred metabolic pathways. As such, trait-based frameworks are independent of taxonomy and promise to reveal otherwise masked interactions and ecological process markers.

Ecological theory helps to explain phenomena, such as process failure. For example, disturbance of the reactor environment, such as sudden changes in sludge quality, will favour the distribution of metabolically flexible organisms; the generalists (Chen et al., 2021). During or after a disturbance, specialists, which are more effective for CH_4_ production, are easily outpaced by generalists. Generalists and specialists co-exist and interact in balance. However, ecological theory states that disturbances create extinction-colonisation events (Prosser et al., 2007). As disturbances increase, the activity of specialists may become increasingly limited. As such, specialists, including methanogens and acetogens, inherently require stable conditions to be competitive or effective (Chen et al., 2021). Perhaps, dynamic management approaches can be developed that introduces disturbance and stability to digesters at certain intervals to increase stability and maintain function.

In addition, changes in nutrient levels in sludge affects the distribution of the types of microbial metabolism, often divided into oligotrophs (slow-growing, adapted to low nutrient concentrations) and copiotrophs (fast-growing, thriving under high nutrient concentrations; Koch, 2001). Oligotrophic microorganisms are thought to have a high affinity for various carbon substrates, hence are considered to be generalists (Poindexter, 1981) that compete for limited resources. Hence, a potential management option resulting from ecological marker discovery is the suppression of foam associated filamentous organisms (known oligotrophs) through specific nutrient amendments.

Metabolic generalists and specialists in anaerobic digesters can be further classified according to their response to disturbance as resistant, resilient or redundant (Allison and Martiny, 2009; Carballa et al., 2015). Resistance means that the microbial composition withstands changes without major variations; resilience defines the ability of the community to change and rebound following a disturbance, while redundancy means that there are functional pathways that can be replaced by a new population, whose function is redundant, thus not affecting system performance. These principles may apply to the whole microbial community or to certain groups of bacteria and may differ in different types of digesters. For example, methanogens in the genus *Methanothrix* (formerly *Methanosaeta*) were found to be resilient to disturbances (e.g., Calderon et al., 2021), while the whole community of methanogens can contain additional (redundant) methanogenic pathways that take over as soon as conditions change (e.g., mixotrophic *Methanosarcina* increasing under ammonia stress, Karakashev et al., 2006). Assessments of how these metrics change, especially for front-end processes such as hydrolysis and fermentation after an imbalance may help to elucidate mechanisms that enhance the stability of the process.

**Figure 6:**
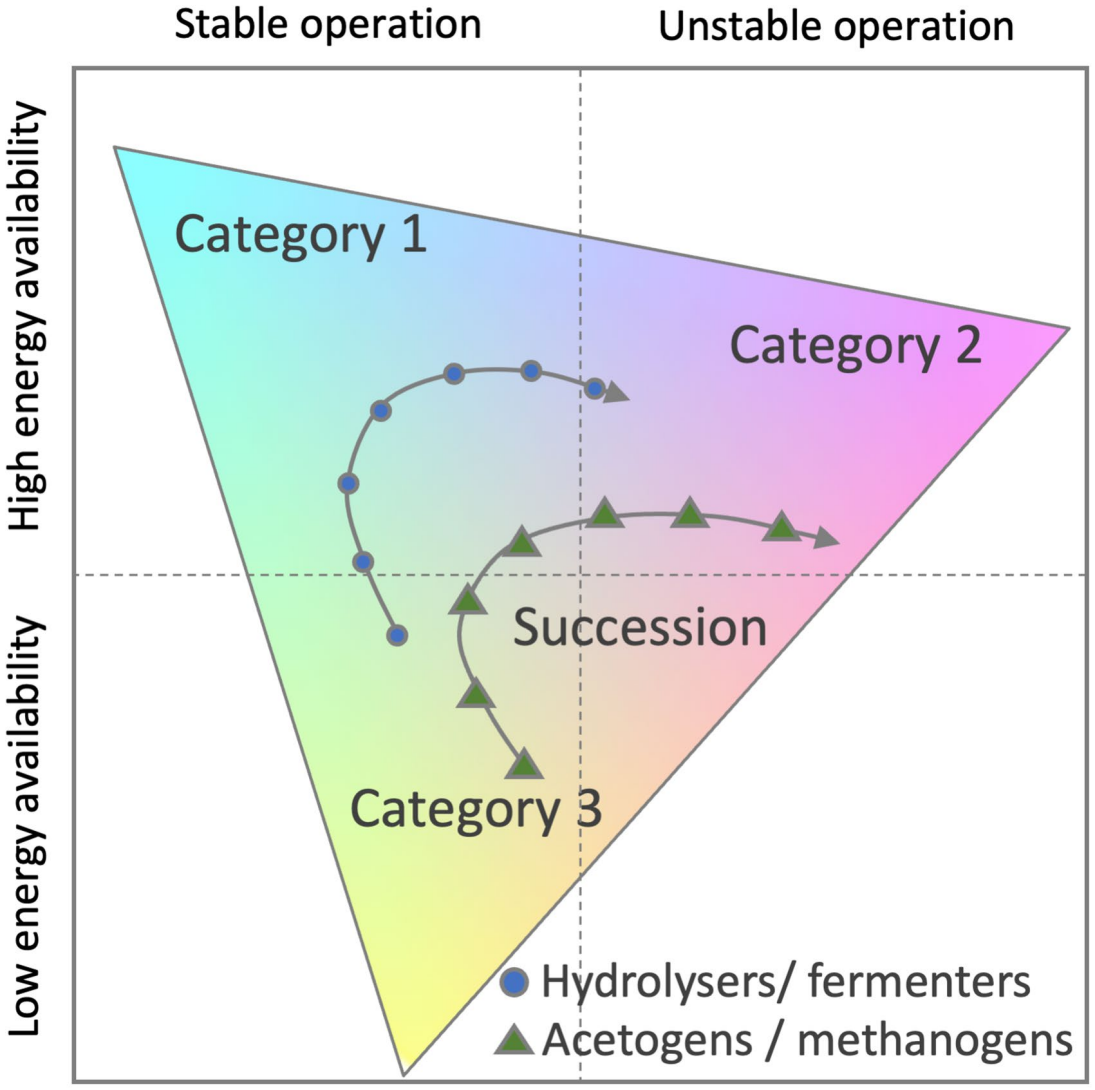
Potential classification scheme for anaerobic digestate microbiome time-series data. Functional composition of each sample is mapped onto three broad life strategy categories (Category 1–3). Category examples can include life strategies such as Competition, Stress and Ruderal traits. Adapted from Bittleston et al., (2021).

However, numerical ecology and its application for modelling related process of the microbial community are evolving. To fully capture the metabolic plasticity of active microbes, microbial ecologists recently proposed to classify successional changes of microbial community traits within a triangular framework (Bittleston et al., 2021). Authors attempted to use the Grime’s competitor-stress-ruderal (C-S-R) framework (Krause et al., 2014; Wood et al., 2018) or the yield-acquisition-stress (Y-A-S) framework (Bittleston et al., 2021). The benefit of these methods is that three classes of genome-based traits can be quantified along three axes within a triangle and successional changes visualised (Figure 6).

In a triangular framework, traits are grouped into three categories and may relate to energy and nutrient acquisition, stress tolerance, chemotaxis or extracellular enzymes, transporters or to damage repair and antibiotic resistance to name a few. Understanding these differences and mapping them to other reactor parameters will lead to tailored biomarkers to prevent problems and increase efficiencies of anaerobic sludge digestion.

### Meta-omics applied to anaerobic digesters

As noted above, a meta-omic (rather than targeted approach) is ideal for discovery of unknowns (particularly relevant to front-end processes). Vanwonterghem et al. (2014b) reviewed the state of art as of 2015 and identified that meta-omic analysis in digesters was highly active, but very much emerging, although this was likely to substantially change over the next decade as more data became available. Increased publicly available data is indeed enhancing the use of metagenomic analysis, with multi-site metastudies now emerging (Campanaro et al., 2020). However genomic information is still largely being used for phylogenetic and genetic discovery, rather than verified functional discovery. This additional step requires the use of detailed metaproteomic (or metatranscriptomic) or metabolomic analysis.

For example, integrating metabolomics or metaproteomic analysis into experimental studies (integrative multiomics) on AD can identify changes in sludge degradability and link observations to active organisms, environmental factors at given time points (Chapleur et al., 2020; Puig-Castellví et al., 2020). Metabolomics adds metabolites to these observations. Furthermore, biochemical ‘fingerprints’ can be created that differentiate sludge samples affected by reactor disturbances or changes in sludge quality during AD over time. Combined with integrative multivariate analyses, any possible association between metabolic fluxes and changes in microbial taxonomy or physicochemical sludge parameters can be investigated.

Metaproteomic analysis in AD is still largely used in a targeted approach, assessing differentiated systems. A particularly early finding with broad applicability is highlighted; that metaproteomic analysis identified relatively low abundance but high expression for methanogens in a carbohydrate fed reactor (Hanreich et al., 2013). It is noted that even for this simple substrate, hydrolysis appeared to be a complex, multi-species activity. This is supported by another lignocellulolytic proteomic study, which highlights the phylogenetic and functional diversity of these systems (Villalobos Solis et al., 2022). In general, metaproteomic analysis of front-end processes appears to be challenged by information overload in highly complex systems, which may benefit from statistical reduction of meta-data. In contrast to metaproteomics, metatranscriptomics is identity focused, and allows ready highlighting of metabolically active (amino acid) organisms (Mei et al., 2020) with more complex function being difficult to determine.

The use of metabolomics is challenging in digesters, due to the complex environment (Vanwonterghem et al., 2014b). To date, there are very few studies that investigate the metabolome of municipal anaerobic digesters. Twelve articles have been identified (see Supplementary Methods for search criteria). The first experimental study that included metabolomics to characterise processes during AD was conducted by a research team in Australia (Beale et al., 2016). In 500 ml stirred glass digesters fed with a mixture of primary sludge and WAS from a municipal WWTP (Melbourne, Australia), the authors investigated how temperature changes and additions of fat, oil and grease materials related to changes in biogas production and taxonomic and metabolic profiles. Using an untargeted approach, the investigators sampled and immediately processed/chemically derivatised aliquots of digester sludge before running it on a GC–MS instrument, identifying approximately 214 metabolites. The authors used partial-least squares discriminant analysis (PLS-DA), which identified 46 metabolites that were associated with the treatments. One fatty acid, capric acid, was clearly up-regulated with the addition of fat, oil and grease materials (Beale et al., 2016). Partial-least squares discriminant analysis is a robust supervised classification method that identifies biologically meaningful clusters in multivariate data based on principle components. Practical guidance on this and similar multivariate analyses techniques for chemical ecology is provided elsewhere (Hervé et al., 2018) and is well implemented; for example, into the R-package mixOmics.^4^

Furthermore, for two additional studies that explored metabolomics on municipal anaerobic sludge, two research teams were working together on the same experiment (Chapleur et al., 2020; Puig-Castellví et al., 2020). In 1 l batch reactors the authors investigated the effects of grass and fish wastes as co-substrates during AD of WAS from a full-scale WWTP (Valenton, France). Using an untargeted approach, metabolites were quantified without prior sample-derivatisation on a LC–MS instrument. The aims of the two studies were to assess changes of WAS degradability and discover links to microbial activity. The authors defined degradability of a sample as the magnitude of change of the metabolic composition. To put simply, the higher the metabolic turnover, the greater material was degraded. Based on that definition, it was found that WAS biodegradability improved by co-digesting fish waste, which was associated with increased degradation of amino acid and biogenic amines (Puig-Castellví et al., 2020). However, biogas quality (methane content) declined together with archaeal diversity, likely due to ammonia inhibition (Chapleur et al., 2020). In contrast, biodegradability of WAS co-digested with grass wastes did not change, which also provided the best biogas production performance (at least 50% grass was needed).

## Concluding remarks

Some authors predict that the ‘exploitation of the microbiome will be a driver of innovation’ for AD in this decade. However, knowledge-gaps in microbial ecology of the front-end metabolism of mesophilic anaerobic digesters limit the desired improvements in stability, methane recovery, risk monitoring, as well as improvements in the management of bulking and foaming.

Importantly, we should not be limited in our interpretation by existing knowledge. What we understand now will change significantly once we start to unravel the reactor microbiome. Ecological markers are important indicators of specific function (including foaming, bulking, and inhibition response), but fundamental improvement in function will require a stronger understanding of the complex environmental interactions. We argue this will require a meta-omic approach focused on discovery, with a metagenomic approach, but utilising particularly metaproteomic and metabolomic methods to unpack function from capability. This will be further enhanced by multi-site studies and publicly available genomic and proteomic data and will have broad applicability outside the digestion sector.

## Author contributions

AB, DB, DD, RS, DO’C, BA, AS, NC, and CR provided concepts and intellectual direction for the review. CK analyzed the data. CK and LK wrote the first draft of the manuscript. All authors contributed to the article and approved the submitted version.

## Funding

This research was funded by an Australian Research Council Industrial Transformation Training Centre Grant (IC190100033).

## Conflict of interest

BA, CR, and NC were employed by the companies South Australian Water Corporation and Melbourne Water Corporation.

The remaining authors declare that the research was conducted in the absence of any commercial or financial relationships that could be construed as a potential conflict of interest.

## Publisher’s note

All claims expressed in this article are solely those of the authors and do not necessarily represent those of their affiliated organizations, or those of the publisher, the editors and the reviewers. Any product that may be evaluated in this article, or claim that may be made by its manufacturer, is not guaranteed or endorsed by the publisher.

## Footnotes

^1^ www.midasfieldguide.org/global

^2^ https://github.com/picrust/picrust2/wiki/Key-Limitations

^3^ https://github.com/egr95/R-codacore

^4^ http://mixomics.org

## Glossary

AD: Anaerobic digestion
ASV: Amplicon sequence variant
BMP: Biochemical methane potential (V g_VS_^−1^)
COD: Chemical oxygen demand
CSTR: Continuous stirred-tank reactors
HTR: Hydraulic retention time
LCFA: Long-chain fatty acid
OFMSW: Organic fraction of municipal solids waste
OLR: Organic loading rate
OTU: Operating taxonomic unit
SRT: Solid retention time
TWAS: Thickened waste activated sludge
VFA: Volatile fatty acids
*VS*: Volatile solids
VSS: Volatile suspended solids
WAS: Waste activated sludge
WWTPs: Wastewater treatment plants
*Acetoclastic/Aceticlastic*: Ability to convert acetate/acetic acid directly to methane. Performed by some methanogens.
*Acetate-oxidation*: A two-step conversion from acetate/acetic acid to hydrogen/carbon dioxide to methane. Performed by bacteria in syntrophic association with methanogens.
*Acidogenic*: Acid-producing.
*Biomarker*: Any biological measurement that is associated with a process of interest. Can be the presence or abundance of a single taxon or enzyme, or combined presence or abundance of a clade or functional pathway. Can include trait-based agglomeration of species or the use of diversity metrics (alpha / beta diversity). Synonym for ecological marker.
*Homoacetogen*: Bacteria whose sole metabolic end-product is acetate. Often capable of growing autotrophically on hydrogen and carbon dioxide or heterotrophically on various carbon compounds.
*Hydrogenotrophic*: The ability to use hydrogen as a source of energy.
*Mixotrophic*: The ability (of methanogens) to utilise both acetate and hydrogen.
*Syntrophic*: The dependence of one species for the metabolic end products of another.
