# Supplementary material for: The role of microbial ecology in improving the performance of anaerobic digestion of sewage sludge
Source: Front Microbiol. 2022 Dec 14;13:1079136. doi: 10.3389/fmicb.2022.1079136 (PMC9801413; doi:10.3389/fmicb.2022.1079136)
Supplement: Supplementary file 1 [file Table_1.DOCX]

**Supplementary Table S1**. Examples of known Bacteria reportedly present in full-scale anaerobic digesters of wastewater treatment plants and some genus-specific information. The expected roles for anaerobic digestion are grouped into four broad categories: Fermentation and hydrolysis (orange), other metabolism (yellow), syntrophy (blue) and foam-associations (green).

| Phylum/Genus | Temperature optimum | A, OA, FA | Gram stain | pH _growth_ | Motility | Genome  size (Mb)^a^ | Genes^a^ | Expected roles for anaerobic digestion | Products | Ref.^c^ |
| --- | --- | --- | --- | --- | --- | --- | --- | --- | --- | --- |
| *Planctomycetota/* ***Planctomyces*** | Mesophiles | FA | - | 6–8 | M, NM | 5.2–8.2 | NA | Fermentation of carbohydrates | Various acids | A, B, C |
| *Chloroflexi/* ***Leptolinea*** | Mesophiles | OA | - | 6.0–7.2 | NM | 3.7–4.4 | 3,301–3,888 | Fermentation of carbohydrates | NA | D, E |
| *Chloroflexi/* ***Candidatus* Brevefilum** | Mesophiles | OA | - | 6.0–8.0 | NM | ~2.57 | ~2,288 | Fermentation of carbohydrates and amino acids, associated with foams | Potentially ethanol, acetate, succinate, propionate, carbon dioxide | D, F, G |
| *Spirochaetota/* ***Spirochaeta*** | Mesophiles & Thermophiles | OA, FA | - | 7–9.4 | M | 2.1–4.7 | 2,928–4,363 | Fermentation of carbohydrates, hydrolysis of sugars | Lactate, acetate, hydrogen, ethanol | A, H |
| *Fermentibacterota/* ***Candidatus* Fermentibacter** | Mesophiles | OA | - | NA | M | 2–2.2 | 1,995–2,349 | Fermentation of carbohydrates | Acetate, hydrogen, hydrogen sulphide | I |
| *Firmicutes/* ***Acetivibrio*** | Mesophiles | OA | - | 6.5–8.6 | M | 3.5–6.2 | 3,036–5,008 | Fermentation of cellulose and carbohydrates, hydrolysis of cellulose | Acetate, ethanol, hydrogen, carbon dioxide, benzoate | J, K, L |
| *Firmicutes/* ***Clostridium*** | Mesophiles | OA, AT | + | 6.5–7.0 | M, NM | NA | NA | Various. Hydrolysis of sugars and proteins, fermentation of carbohydrates, fatty acids, amino acids and other organic compounds, syntrophic oxidation of acetate (*C. ultunense*) | Mixtures of organic acids and alcohols, hydrogen, carbon dioxide | A, K |
| *Firmicutes/* ***Romboutsia*** | Mesophiles | OA | + | 6.5–8.0 | NM | 2.8–3.9 | 2,852–3,535 | Fermentation of carbohydrates | E.g. formate, acetate, lactate | M |
| *Firmicutes/* ***Acetobacterium*** | Mesophiles | OA | + | 7.0–8.0 | M | 2.2–4.1 | NA | Fermentation of carbohydrates and organic acids, acetogenesis | Acetate | N, O |
| *Firmicutes/****Gelria*** | Mesophiles & Thermophiles | OA | + | 5.5–8.0 | NM | NA | NA | Fermentation of organic acids and carbohydrates, potentially syntrophic acetate oxidation with hydrogen-consuming partner, hydrolysis of sugars | E.g. Hydrogen, acetate, propionate, ammonium, bicarbonate | K, P |
| *Bacteroidota/* ***Bacteroides*** | Mesophiles | OA | - | 5.6–9.2 | NM | 5.2–6.3 | ~4,274 | Fermentation of organic acids, hydrolysis of sugars | Succinate, acetate | A, Q |
| *Synergistota/* ***Thermovirga*** | Mesophiles & Thermophiles | OA | - | 6.5–7.0 | M | ~2.0 | ~1,972 | Fermentation of amino acids and organic acids | NA | M, R |
| *Proteobacteria/* ***Dechloromonas*** | Mesophiles | FA | - | NA | M | NA | NA | Organic acid and (per)chlorate decomposition | Chloride | M, S |
| *Proteobacteria/* ***Brachymonas*** | Mesophiles | A, FA | - | 7–7.5 | NM | ~2.5 | ~2,382 | Nitrogen and organic matter decomposition | Nitrogen, carbon dioxide | J, S, T |
| *Proteobacteria/* ***Acidovorax*** | Mesophiles | A, FA | - | NA | M | 4.2–5.5 | NA | Oxidation of organic acids and amino acids | Various acids | M, S, U, V, W |
| *Desulfobacterota/* ***Smithella*** | Mesophiles | OA | - | 6.5–7.5 | M | ~3.23 | ~3,284 | Syntrophic oxidation of fatty acids | Acetate, carbon dioxide, hydrogen | J, S, X, Y |
| *Desulfobacterota/* ***Syntrophobacter*** | Mesophiles | OA | - | 6.0–8.0 | M | ~5 | ~4,100 | Syntrophic oxidation of propionate or fermentation of secondary organic acids | Acetate, hydrogen, carbon dioxide | J, S, Z |
| *Firmicutes/* ***Syntrophomonas*** | Mesophiles | OA | - | 5.5–8.8 | M, NM | 2.94–3.17 | 2,677–3,184 | Syntrophic β-oxidation of fatty acids | Acetate, Propionate, hydrogen | A, K, Y, AA |
| *Firmicutes/*  ***Syntrophaceticus*** | Mesophiles | OA | +, - | 6–8 | NM | NA | NA | Under very low p_H2_: Acetate oxidation, otherwise fermentation of various carbon substrates, e.g. *S. schinkii* | Hydrogen (low p_H2_) Acetate (high p_H2_) | AF |
| *Firmicutes/*  ***Tepidanaerobacter*** | Thermotolerant | OA | + | 4–9.5 | M | NA | NA | Under very low p_H2_: Acetate oxidation, otherwise fermentation of various carbon substrates, e.g. *T. acetatoxydans* | Hydrogen (low p_H2_) Acetate (high p_H2_) | AG |
| *Actinobacteriota/* ***Candidatus* Microthrix** | Psychro- to mesophiles | A^b^ | + | 6.7–8.4 | NM | ~4.2 | ~4,331 | Associated with bulking and foaming; filamentous growth and hydrophobic cells; hydrolysis of lipids and esters; accumulation of long-chained fatty acids | NA | F, AB, AD |
| *Actinobacteriota/* ***Tetrasphaera*** | Mesophiles | A | + | 6.0–9.0 | NM | NA | NA | Foam-associated filamentous cells, accumulation of polyphosphate, hydrolysis of carbohydrates | NA | F, AC |
| *Actinobacteriota/* ***Mycobacterium*** | Mesophiles | A | + | 6.4–7 | NM | 5.6–6.6 | NA | Foam-associated hydrophobic cells | NA | F, AC |

^a^ based on referenced type strains or metagenome assembled genomes; ^b^ was observed to grow anaerobically; ^c^ References for presence in reactors and genus-specific information;

A, aerobic; OA, obligate anaerobe; FA, facultative anaerobe, NM, non-motile; M, motile, Mb, Megabases, NA; not available

Ref, References: A (Yang et al., 2014), B (Andrei et al., 2019), C (Ward et al., 2015), D (Yamada et al., 2006), E (Matsuura et al., 2015), F (Jiang et al., 2021a), G (McIlroy et al., 2017), H (Leschine and Paster, 2015), I (Kirkegaard et al., 2016), J (Nelson et al., 2011), K (Vos et al., 2011), L (Zhang et al., 2018), M (Jiang et al., 2021b), N (Guo et al., 2015), O (Ross et al., 2020), P (Mosbæk et al., 2016), Q (Krieg, 2015), R (DeLong et al., 2014), S (Brenner et al., 2005), T (Laviad et al., 2015), U (Chalupowicz et al., 2020), V, (Ehsani et al., 2015), W (Ide et al., 2019), X (Liu, 1999), Y (Embree et al., 2015), Z (Plugge et al., 2012), AA (Sieber et al., 2010), AB (Rossetti et al., 2005), AC (Goodfellow et al., 2012), AD (Mcilroy et al., 2013), AF (Westerholm et al., 2010), AG (Westerholm et al., 2011)

# References

Andrei, A. Ş., Salcher, M. M., Mehrshad, M., Rychtecký, P., Znachor, P., and Ghai, R. (2019). Niche-directed evolution modulates genome architecture in freshwater Planctomycetes. *ISME J.* 13, 1056–1071. doi: 10.1038/s41396-018-0332-5.

Brenner, D. J., Krieg, N. R., Staley, J. T., and Garrity, G. (2005). Bergey’s manual of systematic bacteriology, Vol 2: The Proteobacteria. *NY Springer*.

Chalupowicz, L., Reuven, M., Dror, O., Sela, N., Burdman, S., and Manulis‐Sasson, S. (2020). Characterization of Acidovorax citrulli strains isolated from solanaceous plants. *Plant Pathol.* 69, 1787–1797.

DeLong, E. F., Lory, S., Stackebrandt, E., and Thompson, F. (2014). *The Prokaryotes: Other Major Lineages of Bacteria and the Archaea*. Springer Berlin Heidelberg.

Ehsani, E., Jauregui, R., Geffers, R., Jarek, M., Boon, N., Pieper, D. H., et al. (2015). First draft genome sequence of the *Acidovorax caeni* sp. nov. type strain R-24608 (DSM 19327). *Genome Announc.* 3, e01378-15. doi: 10.1128/genomeA.01378-15.

Embree, M., Liu, J. K., Al-Bassam, M. M., and Zengler, K. (2015). Networks of energetic and metabolic interactions define dynamics in microbial communities. *Proc. Natl. Acad. Sci. U. S. A.* 112, 15450–15455. doi: 10.1073/pnas.1506034112.

Goodfellow, M., Kämpfer, P., Busse, H.-J., Trujillo, M. E., Suzuki, K., Ludwig, W., et al. (2012). *Bergey’s manual® of systematic bacteriology: Volume five the actinobacteria, part a*. Springer.

Guo, J., Peng, Y., Ni, B. J., Han, X., Fan, L., and Yuan, Z. (2015). Dissecting microbial community structure and methane-producing pathways of a full-scale anaerobic reactor digesting activated sludge from wastewater treatment by metagenomic sequencing. *Microb. Cell Fact.* 14, 1–11. doi: 10.1186/s12934-015-0218-4.

Ide, H., Ishii, K., Fujitani, H., and Tsuneda, S. (2019). Draft genome sequence of Acidovorax sp. strain NB1, isolated from a nitrite-oxidizing enrichment culture. *Microbiol. Resour. Announc.* 8, e00547-19.

Jiang, C., McIlroy, S. J., Qi, R., Petriglieri, F., Yashiro, E., Kondrotaite, Z., et al. (2021a). Identification of microorganisms responsible for foam formation in mesophilic anaerobic digesters treating surplus activated sludge. *Water Res.* 191, 116779. doi: 10.1016/j.watres.2020.116779.

Jiang, C., Peces, M., Andersen, M. H., Kucheryavskiy, S., Nierychlo, M., Yashiro, E., et al. (2021b). Characterizing the growing microorganisms at species level in 46 anaerobic digesters at Danish wastewater treatment plants: A six-year survey on microbial community structure and key drivers. *Water Res.* 193, 116871. doi: 10.1016/j.watres.2021.116871.

Kirkegaard, R. H., Dueholm, M. S., McIlroy, S. J., Nierychlo, M., Karst, S. M., Albertsen, M., et al. (2016). Genomic insights into members of the candidate phylum Hyd24-12 common in mesophilic anaerobic digesters. *ISME J.* 10, 2352–2364. doi: 10.1038/ismej.2016.43.

Krieg, N. R. (2015). “ Bacteroidia class. nov. ,” in *Bergey’s Manual of Systematics of Archaea and Bacteria*, 1–1. doi: 10.1002/9781118960608.cbm00010.

Laviad, S., Lapidus, A., Han, J., Haynes, M., Reddy, T. B. K., Huntemann, M., et al. (2015). High quality draft genome sequence of brachymonas chironomi AIMA4T (DSM 19884T) isolated from a chironomus sp. egg mass. *Stand. Genomic Sci.* 10, 1–7. doi: 10.1186/s40793-015-0010-4.

Leschine, S., and Paster, B. J. (2015). Spirochaeta. *Bergey’s Man. Syst. Archaea Bact.*, 1–18.

Liu, Y. (1999). Characterization of the anaerobic propionate-degrading syntrophs Smithella propionica gen. nov., sp. nov. and Syntrophobacter wolinii. *Int. J. Syst. Bacteriol.* 49, 545–556. doi: 10.1099/00207713-49-2-545.

Matsuura, N., Tourlousse, D. M., Ohashi, A., Hugenholtz, P., and Sekiguchi, Y. (2015). Draft genome sequences of Anaerolinea thermolimosa IMO-1, Bellilinea caldifistulae GOMI-1, Leptolinea tardivitalis YMTK-2, Levilinea saccharolytica KIBI-1, Longilinea arvoryzae KOME-1, previously described as members of the Class Anaerolineae (Chloroflexi. *Genome Announc.* 3, e00975-15. doi: 10.1128/genomeA.00975-15.

McIlroy, S. J., Kirkegaard, R. H., Dueholm, M. S., Fernando, E., Karst, S. M., Albertsen, M., et al. (2017). Culture-independent analyses reveal novel anaerolineaceae as abundant primary fermenters in anaerobic digesters treating waste activated sludge. *Front. Microbiol.* 8, 1134. doi: 10.3389/fmicb.2017.01134.

Mcilroy, S. J., Kristiansen, R., Albertsen, M., Karst, S. M., Rossetti, S., Nielsen, J. L., et al. (2013). Metabolic model for the filamentous “Candidatus Microthrix parvicella” based on genomic and metagenomic analyses. *ISME J.* 7, 1161–1172. doi: 10.1038/ismej.2013.6.

Mosbæk, F., Kjeldal, H., Mulat, D. G., Albertsen, M., Ward, A. J., Feilberg, A., et al. (2016). Identification of syntrophic acetate-oxidizing bacteria in anaerobic digesters by combined protein-based stable isotope probing and metagenomics. *ISME J.* 10, 2405–2418. doi: 10.1038/ismej.2016.39.

Nelson, M. C., Morrison, M., and Yu, Z. (2011). A meta-analysis of the microbial diversity observed in anaerobic digesters. *Bioresour. Technol.* 102, 3730–3739. doi: 10.1016/j.biortech.2010.11.119.

Plugge, C. M., Henstra, A. M., Worm, P., Swarts, D. C., Paulitsch-Fuchs, A. H., Scholten, J. C. M., et al. (2012). Complete genome sequence of Syntrophobacter fumaroxidans strain (MPOBT). *Stand. Genomic Sci.* 7, 91–106. doi: 10.4056/sigs.2996379.

Ross, D. E., Marshall, C. W., Gulliver, D., May, H. D., and Norman, R. S. (2020). Defining genomic and predicted metabolic features of the acetobacterium genus. *mSystems* 5, e00277-20. doi: 10.1128/msystems.00277-20.

Rossetti, S., Tomei, M. C., Nielsen, P. H., and Tandoi, V. (2005). “*Microthrix parvicella*”, a filamentous bacterium causing bulking and foaming in activated sludge systems: A review of current knowledge. *FEMS Microbiol. Rev.* 29, 49–64. doi: 10.1016/j.femsre.2004.09.005.

Sieber, J. R., Sims, D. R., Han, C., Kim, E., Lykidis, A., Lapidus, A. L., et al. (2010). The genome of Syntrophomonas wolfei: new insights into syntrophic metabolism and biohydrogen production. *Environ. Microbiol.* 12, 2289–2301.

Vos, P., Garrity, G., Jones, D., Krieg, N. R., Ludwig, W., Rainey, F. A., et al. (2011). *Bergey’s manual of systematic bacteriology: Volume 3: The Firmicutes*. Springer Science & Business Media.

Ward, N. L., Staley, J. T., and Schmidt, J. M. (2015). Planctomyces. *Bergey’s Man. Syst. Archaea Bact.*, 1–23.

Westerholm, M., Roos, S., and Schnürer, A. (2010). Syntrophaceticus schinkii gen. nov., sp. nov., an anaerobic, syntrophic acetate-oxidizing bacterium isolated from a mesophilic anaerobic filter. *FEMS Microbiol. Lett.* 309, 100–104.

Westerholm, M., Roos, S., and Schnürer, A. (2011). Tepidanaerobacter acetatoxydans sp. nov., an anaerobic, syntrophic acetate-oxidizing bacterium isolated from two ammonium-enriched mesophilic methanogenic processes. *Syst. Appl. Microbiol.* 34, 260–266.

Yamada, T., Sekiguchi, Y., Hanada, S., Imachi, H., Ohashi, A., Harada, H., et al. (2006). Anaerolinea thermolimosa sp. nov., Levilinea saccharolytica gen. nov., sp. nov. and Leptolinea tardivitalis gen. nov., sp. nov., novel filamentous anaerobes, and description of the new classes Anaerolineae classis nov. and Caldilineae classis nov. in the . *Int. J. Syst. Evol. Microbiol.* doi: 10.1099/ijs.0.64169-0.

Yang, Y., Yu, K., Xia, Y., Lau, F. T. K., Tang, D. T. W., Fung, W. C., et al. (2014). Metagenomic analysis of sludge from full-scale anaerobic digesters operated in municipal wastewater treatment plants. *Appl. Microbiol. Biotechnol.* 98, 5709–5718. doi: 10.1007/s00253-014-5648-0.

Zhang, X., Tu, B., Dai, L. R., Lawson, P. A., Zheng, Z. Z., Liu, L. Y., et al. (2018). Petroclostridium xylanilyticum gen. Nov., sp. nov., a xylan-degrading bacterium isolated from an oilfield, and reclassification of clostridial cluster iii members into four novel genera in a new hungateiclostridiaceae fam. nov. *Int. J. Syst. Evol. Microbiol.* 68, 3197–3211. doi: 10.1099/ijsem.0.002966.
