# Supplementary material for: The role of microbial ecology in improving the performance of anaerobic digestion of sewage sludge
Source: Front Microbiol. 2022 Dec 14;13:1079136. doi: 10.3389/fmicb.2022.1079136 (PMC9801413; doi:10.3389/fmicb.2022.1079136)
Supplement: Supplementary file 2 [file Table_2.DOCX]

**Supplementary Table S2**. Common pathogens detected in wastewater sludges.

| **Pathogens** | **Organism name** | **Reference** |
| --- | --- | --- |
| Viruses | Astroviruses, Norwalk viruses, Caliciviruses Reoviruses, Hepatitis viruses Rotaviruses, Enteroviruses  Corona virus (HKU1), Cosavirus, Klassevirus  SARS-CoV-2 | Lewis and Gattie (2002)  Bibby and Peccia (2013)  Foladori et al. (2020) |
| Bacteria | *Legionella* sp., *Aeromonas* sp., *Bacillus* sp., *Listeria* sp., *Brucella* sp., *Campylobacter* sp., *Proteus* sp., *Pseudomonas* sp., *Coxiella* sp., Clostridium sp., Mycobacterium sp., Escherichia sp., Salmonella sp., Shigella sp., Citrobacter sp., Enterobacter sp., Serratia sp.,  Erysipelothrix sp., Staphylococcus sp., Klebsiella sp., Streptococcus sp., Francisella sp., Yersinia sp., and Vibrio sp.  Collinsella aerofaciens, Eubacterium rectale, Streptococcus salivarius, Vibrio mimicus  Bacteroides vulgatus, E. rectale, C. aerofaciens, Streptococcus suis, and S. salivarius  *C. aerofaciens, Arcobacter butzleri, S. salivarius, E. rectale, Acinetobacter johnsonii, S. suis*  *Oligella urethralis, Aeromonas hydrophila, Aeromonas veronii, Mycobacterium smegmatis, Vibrio cholerae, Pseudomonas putida* | Lewis and Gattie (2002)  Li et al. (2022)  Li et al. (2015)  Ju et al. (2016)  Zhang et al. (2021) |
| Nematodes and helminths | Ascaris, Taenia, Hymenolepis, Trichuris, Necator, Toxocara | Lewis and Gattie (2002) |
| Protozoa | Giardia, Cryptosporidium, Entamoeba, Toxoplasma | Benito et al. (2020) |

# References

Benito, M., Menacho, C., Chueca, P., Ormad, M. P., and Goñi, P. (2020). Seeking the reuse of effluents and sludge from conventional wastewater treatment plants: Analysis of the presence of intestinal protozoa and nematode eggs. *J. Environ. Manage.* 261, 110268. doi: 10.1016/j.jenvman.2020.110268.

Bibby, K., and Peccia, J. (2013). Identification of viral pathogen diversity in sewage sludge by metagenome analysis. *Environ. Sci. Technol.* 47, 1945–1951. doi: 10.1021/es305181x.

Foladori, P., Cutrupi, F., Segata, N., Manara, S., Pinto, F., Malpei, F., et al. (2020). SARS-CoV-2 from faeces to wastewater treatment: What do we know? A review. *Sci. Total Environ.* 743, 140444. doi: 10.1016/j.scitotenv.2020.140444.

Ju, F., Li, B., Ma, L., Wang, Y., Huang, D., and Zhang, T. (2016). Antibiotic resistance genes and human bacterial pathogens: Co-occurrence, removal, and enrichment in municipal sewage sludge digesters. *Water Res.* 91, 1–10. doi: 10.1016/j.watres.2015.11.071.

Lewis, D. L., and Gattie, D. K. (2002). Pathogen risks applying sewage sludge to land. *Environ. Sci. Technol.* 36, 286A-293A.

Li, B., Ju, F., Cai, L., and Zhang, T. (2015). Profile and fate of bacterial pathogens in sewage treatment plants revealed by high-throughput metagenomic approach. *Environ. Sci. Technol.* 49, 10492–10502. doi: 10.1021/acs.est.5b02345.

Li, M., Song, G., Liu, R., Huang, X., and Liu, H. (2022). Inactivation and risk control of pathogenic microorganisms in municipal sludge treatment: A review. *Front. Environ. Sci. Eng.* 16, 1–23. doi: 10.1007/s11783-021-1504-5.

Zhang, H., Zhang, Z., Song, J., Cai, L., Yu, Y., and Fang, H. (2021). Foam shares antibiotic resistomes and bacterial pathogens with activated sludge in wastewater treatment plants. *J. Hazard. Mater.* 408, 124855.
