# Supplementary material for: The role of microbial ecology in improving the performance of anaerobic digestion of sewage sludge
Source: Front Microbiol. 2022 Dec 14;13:1079136. doi: 10.3389/fmicb.2022.1079136 (PMC9801413; doi:10.3389/fmicb.2022.1079136)
Supplement: Supplementary file 3 [file Table_3.DOCX]

**Supplementary Table S3.** Reported parameters and contaminants of wastewater sewage sludge.

| **Parameter Concentration Reference** | | | |
| --- | --- | --- | --- |
| Total Solid (TS) |  | 2–9 % | Collivignarelli et al. (2019) |
| Total organic solid |  | 19.8–43.4 % TS |  |
| Total volatile solid |  | 60–85 % TS | Singh and Agrawal (2008) |
| Organic carbon |  | 20.5–40.3 % TS |  |
| Total nitrogen |  | 2.8–4.9 % TS | Gao et al. (2020) |
| Total phosphorus |  | 1.2–3 % TS |  |
| Total potassium |  | <1 % TS | Collivignarelli et al. (2019) |
| **Heavy metals** | | | |
| Fe |  | 2000–38,000 mg/kg | Gao et al. (2020) |
| Mn |  | 100–2621 mg/kg |  |
| Cu |  | 75.8–801 mg/kg |  |
| Zn |  | 300–7500 mg/kg | Khanh Nguyen et al. (2021) |
| Cr |  | 225–900 mg/kg |  |
| Ni |  | 8.6–420 mg/kg |  |
| Pb |  | 30–430 mg/kg | Gao et al. (2020) |
| Cd |  | 0.83–3.0 mg/kg |  |
| Hg |  | 0.1–1.1 mg/kg | Collivignarelli et al. (2019) |
| As |  | 9.9–56.1 mg/kg |  |
| **Organic contaminants** | | | |
| PAHs |  | 0.7–75.2 mg/kg |  |
| PFAS |  | 4.95–980 ng/g | Martins et al. (2016) |
| PCBs |  | 34–137,134 μg/kg |  |
| PhCs and PCPs |  | 0.1–100,000 ng/g | Liu et al. (2018) |
| Microplastic |  | 1500–24,000 particles/g |  |

# References

Collivignarelli, M. C., Canato, M., Abba, A., and Miino, M. C. (2019). Biosolids: what are the different types of reuse? *J. Clean. Prod.* 238, 117844.

Gao, N., Kamran, K., Quan, C., and Williams, P. T. (2020). Thermochemical conversion of sewage sludge: A critical review. *Prog. Energy Combust. Sci.* 79, 100843. doi: 10.1016/j.pecs.2020.100843.

Khanh Nguyen, V., Kumar Chaudhary, D., Hari Dahal, R., Hoang Trinh, N., Kim, J., Chang, S. W., et al. (2021). Review on pretreatment techniques to improve anaerobic digestion of sewage sludge. *Fuel* 285, 119105. doi: 10.1016/j.fuel.2020.119105.

Liu, T., Liu, Z., Zheng, Q., Lang, Q., Xia, Y., Peng, N., et al. (2018). Effect of hydrothermal carbonization on migration and environmental risk of heavy metals in sewage sludge during pyrolysis. *Bioresour. Technol.* 247, 282–290.

Martins, M. N. C., de Souza, V. V., and da Silva Souza, T. (2016). Genotoxic and mutagenic effects of sewage sludge on higher plants. *Ecotoxicol. Environ. Saf.* 124, 489–496.

Singh, R. P., and Agrawal, M. (2008). Potential benefits and risks of land application of sewage sludge. *Waste Manag.* 28, 347–358. doi: 10.1016/j.wasman.2006.12.010.
