# Supplementary material for: The role of microbial ecology in improving the performance of anaerobic digestion of sewage sludge
Source: Front Microbiol. 2022 Dec 14;13:1079136. doi: 10.3389/fmicb.2022.1079136 (PMC9801413; doi:10.3389/fmicb.2022.1079136)
Supplement: Supplementary file 4 [file Data_Sheet_1.DOCX]

**Supplementary Materials and Methods**

## Systematic search for studies of the role of the microbial community in process performance during anaerobic sludge digestion in continuously stirred reactor tanks.

A systematic search was conducted on the Web of Science™️ database (Clarivate Web of Science Core Collection) as part of this review. The database was accessed on 15 December 2021, whereby two sets of search terms were combined. The aim was to assess the availability and number of quality studies that investigate community dynamics during anaerobic digestion in continuously stirred reactor tanks. A total of 68 studies appeared, searching any term related to microbes, together with terms such as '*long-term*', '*anaerobic*', '*digest**', '*continuous*', '*stirred*' and '*wastewater*' or '*sewage*' (see Supplementary Methods for the complete search key) and only 21 of these related to continuously stirred reactors.

Set 1: (((((((((ALL=(microb* AND community )) OR ALL=(microb* AND structure )) OR ALL=(microb* AND composition)) OR ALL=(microb* AND dynamic* AND community)) OR ALL=(microb* AND diversity )) OR ALL=(microb* AND taxonom* )) OR ALL=(microb* AND network* )) OR ALL=(microb* AND interaction*)) OR ALL=(microb* AND genome)) OR ALL=(microb* AND ecology)

Set 2: (((ALL=(temporal OR long-term OR year*)) AND ALL=(anaerobic AND digest* )) AND ALL=(continuous* OR CSTR OR stirred)) AND ALL=(wastewater OR municipal OR sewage OR activated sludge OR mixed liquor)

The search 'Set 1' AND 'Set 2' resulted in a total of 68 studies, which were inspected and filtered manually according to the following selection criteria:

Only studies were selected that used continuously stirred reactor tanks for their investigations.

Only studies were selected that, either investigated full-scale plants or used sewage sludge (primary or secondary or mixed) that was sampled from a full-scale plant to feed laboratory scale reactors.

All other studies were removed.

## Literature search for studies that investigate the metabolome of municipal anaerobic digesters.

Twelve articles have been identified based on a search in the Web of Science™️ database (Clarivate Web of Science Core Collection, accessed 03 Aug 2022) with the search terms "(ALL=(metabolom* AND anaerobic digest*)) AND ALL=(wastewater OR sewage*)".

## Details of data processing of publicly available amplicon sequences (accession number PRJNA645373)

102 fastq files containing raw amplicon sequences (51 bacterial and 51 archaeal amplicons) were downloaded from the National Center for Biotechnology Information (NCBI), which was made available by by Jiang et al. (2021) from the Center for Microbial Communities at Aalborg University, Denmark.

- Archaeal primers used were 340F (CCCTAHGGGGYGCASCA) and 915R (GWGCYCCCCCGYCAATTC) - V3–V5
- Bacterial primers used were 27F (AGAGTTTGATCCTGGCTCAG) and 534R (ATTACCGCGGCTGCTGG) - V1–V3

All 102 fastq files were imported to Qiime 2 (v 2021.8), primer-trimmed with cutadapt and denoised with DADA2, before taxonomic assignments (qiime feature-classifier classify-sklearn) using a classifier trained with the publicly available MiDAS taxonomy and sequences (MiDAS4.8.1.fa and MiDAS.4.8.1.qza) (www.midasfieldguide.org/guide/downloads). For comparison, taxonomy was assigned using a Silva classifier (Silva-138-99). Phylogenetic trees for bacteria and archaea were created with 'qiime fragment-insertion sepp', using the 'sepp-refs-silva-128.qza' as a reference tree for both, bacteria and archaea.

For DADA2, the bacterial forward and reverse reads were truncated at 300 and 259 bases and the maximal expected error was 4 and 5, respectively. Forward and reverse reads were paired. On the other hand, only the forward reads were processed for archaeal amplicons, truncated at 229 bases with a maximum expected error of 2. This resulted in 1,299,791 (4,218 ASVs) and 2,313,012 (932 ASVs) bacterial reads and archaeal reads respectively.

The authors sampled sludge from 16 anaerobic Digesters in Denmark to identify foam-associated prokaryotes. Using the same sludge but for a separate experiment, the same group has developed a foaming test and sampled/extracted the sludge DNA from the bubble layer during the foaming tests to compare the bubble layer with digester sludge (Jiang et al., 2018, 2021). Hence, 76 files of the 102 files (38 bacterial and 38 archaeal sequences), related to the foaming-test experiment and the remaining samples related to reactor comparisons. For further analysis we only considered the 76 samples. These amplicon sequences are the result of 2 sets of samples (bubble layer and normal digester sludge) from a total of 19 digesters from 11 wastewater treatment plants.

76 samples = 19 digesters x 2 sample types (bubble layer and digester sludge) x 2 primers (bacteria and archaea).

R Studio and the package phyloseq was used for all subsequent analyses. Custom script was used to merge the taxonomy of MiDAS and Silva to create a new taxonomy table that replaces rows in the Midas database with the Silva taxonomy, when the MiDAS taxonomy contained no values for phylum, class, order, family, genus or species in the MiDAS taxonomy (but was present in the Silva taxonomy).

Principle coordinate Analysis (PcoA) was performed separately for Bacteria and Archaea with 'phyloseq::ordinate' using weighted and unweighted UniFrac distances. The ordination was visualised with ggplot on two axes.

Phylofactor analysis (Washburne et al., 2019) was performed on the bacterial phylogenetic tree, using the function 'PhyloFactor' with Location (of wastewater treatment plant) as the explanatory variable and aggregated relative abundances in the tree (Isometric log ratios) as the response. Three factors (1, 3 and 5) were selected for further visualisations in ggtree (Yu et al., 2017).

# References

Jiang, C., McIlroy, S. J., Qi, R., Petriglieri, F., Yashiro, E., Kondrotaite, Z., et al. (2021). Identification of microorganisms responsible for foam formation in mesophilic anaerobic digesters treating surplus activated sludge. *Water Res.* 191, 116779. doi: 10.1016/j.watres.2020.116779.

Jiang, C., Qi, R., Hao, L., McIlroy, S. J., and Nielsen, P. H. (2018). Monitoring foaming potential in anaerobic digesters. *Waste Manag.* 75, 280–288. doi: 10.1016/j.wasman.2018.02.021.

Washburne, A. D., Silverman, J. D., Morton, J. T., Becker, D. J., Crowley, D., Mukherjee, S., et al. (2019). Phylofactorization: a graph partitioning algorithm to identify phylogenetic scales of ecological data. *Ecol. Monogr.* 89, e01353. doi: 10.1002/ecm.1353.

Yu, G., Smith, D. K., Zhu, H., Guan, Y., and Lam, T. T. (2017). ggtree: an R package for visualization and annotation of phylogenetic trees with their covariates and other associated data. *Methods Ecol. Evol.* 8, 28–36.
